# Genetic architecture of fusarium head blight disease resistance and associated traits in Nordic spring wheat

**DOI:** 10.1007/s00122-022-04109-9

**Published:** 2022-05-21

**Authors:** Vinay Kumar Reddy Nannuru, Susanne S. Windju, Tatiana Belova, Jon Arne Dieseth, Muath Alsheikh, Yanhong Dong, Curt A. McCartney, Maria Antonia Henriques, Hermann Buerstmayr, Sebastian Michel, Theodorus H. E. Meuwissen, Morten Lillemo

**Affiliations:** 1 Department of Plant Sciences, Norwegian University of Life Sciences, 1432 Ås, Norway; 2 Graminor AS, 2322 Ridabu, Norway; 3 Centre for Molecular Medicine Norway, Faculty of Medicine, University of Oslo, 0318 Blindern, Norway; 4 Department of Plant Pathology, University of Minnesota, St. Paul, MN 55108 USA; 5 Morden Research and Development Centre, Agriculture and Agri-Food Canada, Morden, MB Canada; 6 Institute of Biotechnology in Plant Production, Department of Agrobiotechnology, University of Natural Resources and Life Sciences Vienna, 3430 Tulln, Austria; 7 Department of Animal and Aquacultural Sciences, Norwegian University of Life Sciences, 1432 Ås, Norway; 8 Present Address: Department of Plant Science, University of Manitoba, Winnipeg, MB R3T 2N2 Canada

## Abstract

**Key message:**

This study identified a significant number of QTL that are associated with FHB disease resistance in NMBU spring wheat panel by conducting genome-wide association study.

**Abstract:**

Fusarium head blight (FHB) is a widely known devastating disease of wheat caused by *Fusarium graminearum* and other *Fusarium* species. FHB resistance is quantitative, highly complex and divided into several resistance types. Quantitative trait loci (QTL) that are effective against several of the resistance types give valuable contributions to resistance breeding. A spring wheat panel of 300 cultivars and breeding lines of Nordic and exotic origins was tested in artificially inoculated field trials and subjected to visual FHB assessment in the years 2013–2015, 2019 and 2020. Deoxynivalenol (DON) content was measured on harvested grain samples, and anther extrusion (AE) was assessed in separate trials. Principal component analysis based on 35 and 25 K SNP arrays revealed the existence of two subgroups, dividing the panel into European and exotic lines. We employed a genome-wide association study to detect QTL associated with FHB traits and identify marker–trait associations that consistently influenced FHB resistance. A total of thirteen QTL were identified showing consistent effects across FHB resistance traits and environments. Haplotype analysis revealed a highly significant QTL on 7A, *Qfhb.nmbu.7A.2,* which was further validated on an independent set of breeding lines. Breeder-friendly KASP markers were developed for this QTL that can be used in marker-assisted selection. The lines in the wheat panel harbored from zero to five resistance alleles, and allele stacking showed that resistance can be significantly increased by combining several of these resistance alleles. This information enhances breeders´ possibilities for genomic prediction and to breed cultivars with improved FHB resistance.

**Supplementary Information:**

The online version contains supplementary material available at 10.1007/s00122-022-04109-9.

## Introduction

Wheat (*Triticum aestivum* L.) is one of the most widely cultivated crops with around 214 million hectares and a global production of 734 million tonnes in 2018 (FAOSTAT 2018). It is also the most cultivated cereal crop in Europe (FAOSTAT 2018). To meet the increasing demand for food and feed, breeders are continuously developing novel, more efficient germplasm with improved yield, quality and disease resistance. To lower the risk of crop losses from plant diseases and to reduce the dependency on pesticides and their environmental impact, new cultivars are needed that combine excellent disease resistance with productivity and end-use quality. Fusarium head blight (FHB) is a devastating fungal disease affecting the wheat production worldwide. FHB can cause severe yield losses due to failed kernel development or because infected kernels are shriveled, discolored and low in test weight (McMullen et al. 2012). *Fusarium graminearum,* which produces the mycotoxin deoxynivalenol (DON) is found to be the most causal agent of FHB in wheat (McMullen et al. 1997; Goswami and Kistler 2004; Hofgaard et al. 2016). Mycotoxins, such as DON, may cause severe problems and is a threat to both animals and humans, reaching from feed refusal and poor weight gain in animals to immunological problems in humans (McMullen et al. 2012). Threshold levels of DON concentration set by the European Union range between 200 µg/kg for processed cereal-based foods and baby foods for infants and young children and 750 µg/kg for cereals intended for direct human consumption and cereal flour. For unprocessed cereals, the threshold has been set to 1250 µg/kg (European Commission 2006).

Breeding for disease resistance is the most cost-effective method to control this disease (Buerstmayr et al. 2002). To develop resistant cultivars, proper understanding of resistance mechanisms is required. Resistance to FHB has been divided into active and passive resistance mechanisms. The active mechanisms are commonly divided into five different types: Type I: Resistance to invasion (initial infection), Type II: Resistance to pathogen spread in infected tissue, Type III: Resistance to toxin accumulation (Miller et al. 1985), Type IV: Resistance to kernel infection and Type V: Tolerance (Schroeder and Christensen 1963; Miller et al. 1985; David Miller and Arnison 1986; Mesterhazy 1989, 1995; Mesterházy et al. 1999; Buerstmayr and Lemmens 2015). Further, the following passive resistance mechanisms have been suggested: Type I: plant height, Type II: presence/absence of awns, where the presence of awns increases the disease severity and otherwise, Type III: spikelet density within the head, and Type IV: escape, flowering in boot stage and the ability of florets to extrude anthers (Mesterhazy 1995). The different resistance mechanisms are quantitative (complex) in nature and highly influenced by the environment, making breeding for resistance by traditional ways difficult. In addition, due to incomplete understanding of factors that influence the disease development and difficulty in efficient application, the use of fungicides for controlling FHB is limited (McMullen et al. 1997; Goswami and Kistler 2004).

New marker technologies, enabling quantitative trait loci (QTL) detection, association mapping and subsequently marker detection, have the potential to increase the efficacy of resistance breeding for FHB and in addition dissect and enhance the understanding of the genetic basis of the complex resistance mechanisms.

Many QTL mapping studies for FHB resistance in wheat have been performed over the past two decades, and with the single-nucleotide polymorphism (SNP) marker technology, more markers are available, and it is possible to map resistance QTL more precisely in linkage maps and physical maps. These studies have been performed using different biparental populations and have revealed chromosomal regions harboring FHB resistance loci. More than a decade ago, Buerstmayr et al. (2009) and Liu et al (2009) compared and assembled the information from several of these studies in maps displaying interesting chromosomal regions harboring FHB resistance QTL, which can be further tested and potentially utilized in resistance breeding. The possibility to map the resistance QTL has also led to a closer understanding of genes and mechanisms underlying the resistance traits. The most researched QTL for FHB resistance in wheat is *Fhb1* on chromosome arm 3BS, which is derived from the Chinese wheat cultivar Sumai 3 (Waldron et al. 1999) and has been detected in many mapping studies (Buerstmayr et al. 2009; Liu et al. 2009). It confers mainly Type II resistance (Bai et al. 2018) and can reduce disease severities by 20–50% (Bernardo et al. 2012; Jin et al. 2013). The gene was recently cloned in two independent studies and shown to encode a histidine-rich calcium-binding protein (Li et al. 2019; Su et al. 2019).

In recent years, genome-wide association studies (GWAS) have also been applied for detection of FHB resistance QTL. The benefit of these studies is the ability to capture historic recombination events and utilize collections with a wide genetic background. This increases the possibility to detect interesting QTL for resistance directly in breeding relevant germplasm and enables a targeted incorporation of resistance QTL into breeding programs. Some of these studies have demonstrated both plant height (PH) and anther extrusion (AE) to be negatively correlated with FHB severity. Skinnes et al. (2010) detected a consistent and negative correlation between AE and FHB disease severity, and AE and DON content in the Arina x NK93604 mapping population. A study by Lu et al. (2013) performed on the mapping population Shanghai3/Catbird x Naxos confirmed the negative correlation between AE and FHB, and the QTL analysis further confirmed the relationship; eight out of ten AE QTL detected in the study coincided with FHB severity. Kubo et al. (2013) demonstrated that partially extruded anthers were a good source for FHB infection, while rapid extrusion and ejection of the anthers contributed to the avoidance of infection by FHB. A meta-analysis performed by Mao et al. (2010) confirmed a negative association between PH and FHB, where coincident QTL for PH and FHB were detected on chromosomes 2D, 3A, 4B, 4D and 7A.

QTL for both FHB disease severity and DON content can serve as valuable sources for disease resistance breeding in wheat. SNP markers closely linked to the resistance QTL can be further tested and used in resistance breeding for FHB and DON resistance.

The aim of this study was to identify QTL for both FHB disease severity and DON in a diverse panel of 300 Nordic spring wheat lines by GWAS, to study the consistency of these QTL across environments and their association with AE and then, to further validate these QTL to observe the potential enhancement of genomic prediction models for FHB disease resistance.

## Material and methods

### Plant material

The germplasm used in the study was a collection of 300 hexaploid spring wheat accessions including 186 lines from Norway, 40 lines from Sweden, 37 from CIMMYT and some additional accessions from Australia, Brazil, Canada, China, Czech Republic, Denmark, Finland, France, Germany, Japan, the Netherlands, Poland. Russia, Slovakia, South Africa, Switzerland, UK and USA. This panel is hereafter referred to as the NMBU spring wheat panel (Online resource 1). The NMBU spring wheat panel is further grouped into European and exotic lines based on population structure (see section “Results”). In the following, complete panel refers to the whole NMBU spring wheat panel and European panel refers to the subset of lines with European origin.

As a validation panel, an independent set of 358 new breeding lines from the commercial spring wheat breeding program of Graminor was used to validate QTL identified in the NMBU spring wheat panel. This set of lines is hereafter referred to as the validation panel (Online resource 1).

### Fusarium field design, inoculation and scoring

#### Field trials in Norway

The NMBU spring wheat panel was planted in α-lattice designs with two replicates at two locations in Norway: Vollebekk research farm at the Norwegian University of Life Sciences, Ås (59°N, 90 m above sea level) in 2013, 2014, 2015, 2019, and Staur research farm close to Hamar (60°N, 153 m above sea level) in 2014 and 2015. To ensure a high Fusarium disease pressure in these trials, spawn inoculation of the fields was performed. Grain spawn, oat kernels infected with *F. graminearum*, was prepared and distributed in the field based on a modified protocol from Dr. Bernd Rodemann, Julius Kuhn Institute, Braunschweig using a mixture of four isolates as described by Lu et al. (2013) and Tekle et al. (2018). Mist irrigation was applied every 10–15 min per hour from 19:00 to 23:00 every evening in the period starting from spawn inoculation at the booting stage to 3–4 weeks after flowering to ensure high disease pressure.

FHB disease assessments were performed at the beginning of maturity, when the stems of the plants in the individual plots just started to turn yellow, but the heads were still green. At Staur research farm five random heads at three different positions in each plot were evaluated. At Vollebekk research station ten random heads at two different positions in each plot were evaluated. The evaluation was performed visually by counting the number of *Fusarium*-infected spikelets and dividing this by the total number of spikelets giving a percentage of infected spikelets in each plot. The field plots were harvested with a plot combine, and the level of DON in each sample was evaluated by GC–MS at the University of Minnesota mycotoxin diagnostic laboratory (Mirocha et al. 1998). Days to heading (DH) was scored in the same field as the Fusarium disease evaluation in every testing environment at the time when 50% of the heads in the plot had emerged. PH was measured in centimeter in the Fusarium nurseries at Vollebekk (in 2013, 2014, 2015 and 2019) and Staur (in 2014 and 2015).

AE was evaluated at Vollebekk in both the greenhouse (2013) and small field plots (2013) and in hill plots (2014, 2019 and 2020), and at Staur in hill plots (2014 and 2015) in different nurseries adjacent to the Fusarium disease assessment field, avoiding the confounding effects of mist irrigation on the AE assessment. AE was assessed visually by a scale from 0 to 9, where 0 represents no anther extrusion and 9 represents full anther extrusion as described by Skinnes et al. (2010).

#### Field trial in Canada

A field trial was also conducted in Canada at Morden, Manitoba, in the year 2019. The lines were planted in a single 1-m-long row using a six-row cassette Row XL, Wintersteiger planter following an alpha-lattice design with two replicates. *F. graminearum* corn kernel inoculum was used. The inoculum was prepared using four *F. graminearum* isolates (HSW-15-39 (3-ADON), HSW-15-87 (3-ADON), HSW-15-27 (15-ADON) and HSW-15-57 (15-ADON) from Dr. Henriquez’s culture collection, modifying the protocol of Gilbert and Woods (2006). Each isolate was inoculated in individual pans containing sterile corn and incubated for one month. The inoculum was dispersed at a rate of 8 g per row at 4–5 leaf stage. The inoculum application was followed by irrigation three times a week (Monday, Wednesday and Friday) using Cadman Irrigations travelers with Briggs booms. Infected rows were rated around 21 days after anthesis for incidence and severity in the field. The incidence was estimated with visual assessment using a scale from 0 to 100% (percent of heads with infection per plot), while the severity was estimated with visual assessment of the average amount of infection on infected heads per plot using a scale from 0 to 100% (Stack and McMullen 1995). The FHB index is the product of Incidence × Severity divided by 100. PH was measured in cm from ground to top of heads excluding the awns. After harvest (using low wind speed on the combine to retain lightweight Fusarium-damaged kernels (FDK), samples were cleaned and a minimum of 10 g of well-mixed seed were ground to flour. From each replicate, 1.000 g of flour was used for DON (ppm) analysis using ELISA tests.

#### Field trial in Austria

A subset of 200 lines from the NMBU spring wheat panel was tested in a field trial conducted at the experimental station of the Department of Agrobiotechnology, Tulln in 2020 (9°N, 177 m above sea level). The trial was laid out as a randomized complete block design with two replicates and inoculated with the DON-producing *Fusarium culmorum* isolate Fc91015. Spray inoculations of each individual plot were performed with a backpack sprayer when 50% of the plants within this plot reached the flowering stage. FHB symptoms were visually scored on a plot basis as the percentage of infected spikelets 14, 18, 22 and 26 days after the particular plot reached the flowering stage. Anther retention (AR) was recorded with a scale of 0–20, where four central florets on five normal main spikes per plot were manually opened and the number of florets with at least one anther inside or trapped between palea and lemma were counted. This was converted to AE with the following formula: AE = [1 − (AR 0 − 20)/20] × 9. Days to anthesis (DA) was recorded in this field trial and was used instead of DH, to correct the FHB and DON measurements for confounding effects of earliness. PH within each plot was measured in centimeter at the end of the cropping season (BBCH 89–92).

#### Field trials of the validation panel

The validation panel of new breeding lines was evaluated in a spawn-inoculated disease nursery with two replicates at Vollebekk research station in 2020, following the same methodology as described for the NMBU spring wheat panel above. Data were obtained for the traits DH, PH, FHB and DON. In addition, AE was assessed in an adjacent hill plot nursery with two replicates following the same methodology as described above for the NMBU spring wheat panel.

The validation panel was also evaluated with two replicates at the experimental station of the Department of Agrobiotechnology, Tulln in 2021 in Austria. Data were obtained for the traits DA, PH, FHB and AR. However, data for DON were not obtained from this trial. AR was converted to AE with the formula mentioned above.

### Phenotypic statistical analysis

The raw data from the DON level assessment were transformed by log_10_ (DON level + 1) to approximately obtain normally distributed values that were used throughout the entire analyses. Least square means of FHB disease severity, DON, DH, PH and AE for single environments (= trials) and across different environment for each trait were calculated using the “lme4” package (Bates et al. 2015) and “lmerTEST” (Kuznetsova et al. 2017) in R (Team 2021) using the following models:

1 $$P_{ikn} = \mu + g_{i} + B_{k} + R_{n} + R:B_{kn} + e_{ikn}$$

and

2 $$P_{ijkn} = \mu + g_{i} + E_{j} + g \times E_{ij} + B_{k} + R_{n} + R:B_{kn} + e_{iJkn} { }$$

From the above models, model 1 was used to calculate LSmeans of single environments for each trait, where $P_{ikn}$ is the phenotype (trait value) of the *i*th variety in the *n*th replicate in the *k*^th^ block. $\mu$ is the general mean, $g_{i}$ is the fixed effect of the *i*th variety, $B_{k}$ is the random effect of *k*th block, $R_{n}$ is the random effect of *n*th replicate, $R:B_{kn}$ is the random effect of the *k*th block within *n*th replicate, and $e_{ikn}$ is the error term. And model 2 was used to calculate LSmeans across the different environments tested for each trait, where $P_{ijkn}$ is the phenotype (trait value) of the *i*th variety in the *n*th replicate in the *k*th block in *j*th environment. $\mu$ is the general mean, $g_{i}$ is the fixed effect of the *i*th variety, $E_{j}$ is the random effect of the *j*th environment, $g \times E_{ij}$ is the random effect of the *i*th variety grown under *j*th environment (interaction), $B_{k}$ is the random effect of *k*th block, $R_{n}$ is the random effect of *n*th replicate, $R:B_{kn}$ is the random effect of the *k*th block within *n*th replicate, and $e_{ijkn}$ is the error term.

FHB scorings and DON content can be confounded with variation in PH and DH, to adjust for these confounding effects, regressions of FHB and DON on PH and DH was performed and the residuals used for the further analysis, while in the case of the trials at Morden and Tulln DA was used instead of DH. The residuals are FHB corrected for PH and DH/DA (FHBcPHDH), and DON corrected for PH and DH/DA (DONcPHDH).

Pearson correlations between the traits were calculated by Pearson method (Benesty et al. 2009) in R. Principal component analysis (PCA) was performed on the available phenotypic data of FHBcPHDH, DONcPHDH and AE from all the tested environments to determine the principal coordinate values (PC scores) for each of the above-mentioned traits using the R package “Factoextra” (Kassambara and Mundt 2017).

Broad-sense heritabilities were calculated for the across environment means of FHB, DON and AE based on the variance component estimates (Genetic Variance and Residual Variance) obtained from the R package “Heritability” (Kruijer et al. 2015) in the complete panel and in the European panel using the formula:

$$H^{2} = \frac{{\sigma_{g}^{2} }}{{\sigma_{g}^{2} + \sigma_{E}^{2} }}$$

In the above formula, $\sigma_{g}^{2}$ is genetic variance, and $\sigma_{E}^{2}$ is the error variance.

### Genotypic data

Seedlings of the NMBU spring wheat panel were grown in the greenhouse and genomic DNA was extracted from fresh young leaves using the DNeasy plant DNA extraction kit (Qiagen). The lines were genotyped with the 35 K Axiom^®^ array (Allen et al. 2017), Trait Genetics Illumina 25 K SNP Chip, and in addition, some KASP and SSR markers for key agronomic and disease resistance traits (Rasheed et al. 2016) were also included in our study. The SSR markers were converted to biallelic state. Both the KASP and SSR markers were initially assigned to a fictive chromosome in the 35 K and 25 K genotypic datasets and later the markers which showed significant association with the traits were approximately placed close to the relative chromosomal position on the consensus map, based on physical map positions obtained from the wheat reference genome IWGSC RefSeq v1.0 (Appels et al. 2018) and LD with other significant markers. “MapChart 2.32.” (Voorrips 2002) was used for graphical representation of QTL positions with their intervals.

Markers were filtered based on 10% missing data and minor allele frequency of ≥ 5% in the lines. Heterozygous genotypes were regarded as missing data. Positional information was assigned using the consensus 35 K SNP map (Allen et al. 2017) and Trait Genetics Illumina 25 K SNP Chip. After filtering and removing redundant markers, in total 14,713 and 21,652 markers remained for the association mapping in 35 and 25 K genotypic datasets (Online resource 1).

The validation panel was genotyped using Trait Genetics Illumina 25 K SNP Chip. After genotyping, the markers were filtered based on the criteria mentioned above. For the present study, marker data were available for only 131 of the lines in the validation panel. The most significant SNP markers of the *Qfhb.nmbu.7A.2* region were converted to KASP markers, which were used for validation purpose in the study. Primer sequences were designed using the online PolyMarker tool (http://www.polymarker.info/) (Ramirez-Gonzalez et al. 2015) or downloaded directly from CerealsDB (https://www.cerealsdb.uk.net/cerealgenomics/CerealsDB/).

### Linkage disequilibrium and population structure

Calculation of the linkage disequilibrium (LD) over the entire genome was performed using the software TASSEL 5 (Bradbury et al. 2007). The LD was calculated with every mapped marker with allele frequencies > 0.05, over the 21 chromosomes, in a sliding window approach, with 1000 as the window size. Squared allele frequency correlation *r*^*2*^ (Hill and Weir 1988) was used to calculate pair-wise LD. The p-values were calculated using Fisher’s test in TASSEL, and a threshold of *p* < 0.001 was used for detecting significant LD between markers. The average genome-wide LD decay was visualized by plotting all intra-chromosomal *r*^*2*^ values of all chromosomes against genetic distance in cM between the marker pairs. To summarize the relationship between the LD decay and genetic distance, a nonlinear model described by Marroni et al. (2011) was used. For further information on the method of LD calculation, refer to Ruud et al. (2019).

### Genome-wide association mapping

Association analysis was performed on the traits AE, FHBcPHDH and DONcPHDH for all the tested environments, and across environment means and PC scores of each trait using the “GAPIT” R package (Lipka et al. 2012). In our study, we tested several methods for GWAS, such as mixed linear model (Yu et al. 2006), compressed MLM (Zhang et al. 2010), enriched compressed MLM (Li et al. 2014) and FarmCPU (Liu et al. 2016). Association mapping was performed separately for the total population of 300 spring wheat lines and for the European subpopulation consisting of 237 lines using both genotype datasets (35 and 25 K).

Since FHB resistance is a highly quantitative trait with mostly small-effect loci (Bai et al. 2018), the Bonferroni correction or false discovery rate (FDR) would be too strict criteria for identifying QTL (Haikka et al. 2020). However, in this study we decided to use FDR at a rate of 5% for across environment means of each trait. And the QTL were considered significant at FDR < 5%. In addition, we evaluated how often these significant QTL were detected with a less stringent threshold of − log10 (*p*) > 3.0 in the different environments. Those markers which were consistently detected above this threshold in two or more tested environments for a particular QTL region were considered as robust associated markers and later used for haplotype analysis and allele stacking. Quantile–quantile (QQ) plots were inspected to identify the level at which the observed p-values started to deviate from the expected values under the null hypothesis, and for the presence of spurious associations.

### Haplotype analysis and allele stacking

QTL that were consistently detected in different environments were further investigated to build haplotypes of common QTL for the traits FHBcPHDH, DONcPHDH and AE. Associated markers in those consistent QTL regions were used to construct haplotypes provided they were significant, consistent across the environments and having good LD with the highest significant marker in the QTL region. These haplotypes were tested with across environment means of corrected FHB disease severity, corrected DON content and AE from the complete panel to assess which haplotype contributes to resistance. For this purpose, pair-wise comparisons by Mann–Whitney–Wilcoxon test (Mann and Whitney 1947) were conducted in the European and complete panel. Haplotype analysis was also carried out in the validation panel of new breeding lines from Graminor to validate the effect in an independent dataset from which the haplotypes were constructed by using the same QTL detected in the NMBU spring wheat panel (25 K).

Additionally, a validation was done on the haplotypes constructed based on KASP markers from the QTL *Qfhb.nmbu.7A.2*. These haplotypes were tested with across environment means of corrected FHB disease severity, corrected DON content and AE from the complete panel to assess which haplotype contributes to resistance.

For allele stacking, markers which were highly significant in different QTL regions were chosen for FHBcPHDH, DONcPHDH and AE. The resistant allele was determined based on the predicted allele effect of the markers associated with the QTL. Then, the lines were grouped based on the number of resistance alleles they contained. Mann–Whitney–Wilcoxon test (Mann and Whitney 1947) was used to compare the significance of differences between the groups.

RStudio (Team 2021) was used to visualize the graphs using the following packages: “ggplot2” (Wickham et al. 2016) and “ggpubr” (Kassambara and Kassambara 2020).

## Results

### Phenotypic data analysis

**Fig. 1:**
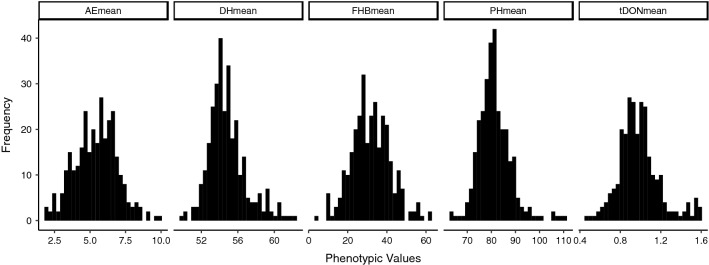
Histogram distributions based on the mean phenotypic data over all testing environments for AE, DH, FHB, PH and tDON for the NMBU spring wheat panel

Across environment means of FHB severity, DON, AE, PH and DH showed continuous variation that resembled normal distributions (Fig. 1). Considerable variation was observed within the complete panel and the European panel for all the traits in all the tested environments (Supplementary Fig. 1).

The FHB severity was positively correlated with DON with high significance (*r* = 0.66, *p* < 0.0001) and negatively correlated with PH (*r* = − 0.43, *p* < 0.0001) and AE (*r* = − 0.48, *p* < 0.0001). DON was positively correlated with DH (*r* = 0.38, *p* < 0.0001) and negatively correlated with PH (*r* = − 0.31, *p* < 0.0001) and AE (*r* = − 0.47, *p* < 0.0001) (Table 1).

**Table 1:** Pearson’s correlation coefficients between FHB severity, DON content, anther extrusion (AE), plant height (PH) and days to heading (DH) for across environment means (LSmeans) of the NMBU spring wheat panel

	AE mean	DH mean	FHB mean	PH mean
DH mean	0.054	–		
FHB mean	− 0.48***	− 0.013	–	
PH mean	0.16**	0.0013	− 0.43***	–
DON mean	− 0.47***	0.38***	0.66***	− 0.31***

*P < 0.05

**P < 0.001

***P < 0.0001

Estimated heritabilities (*H*^2^) of across environment means of FHB, DON and AE were 0.50, 0.69 and 0.74 in the complete panel and 0.34, 0.55 and 0.72 for the European panel respectively.

Phenotypic data for the main current spring wheat varieties in Norway as well as selected resistance sources in the NMBU spring wheat panel are shown in Table 2. Among the market varieties, the more recent varieties (year of release in parentheses) Mirakel (2012), Seniorita (2014) and Caress (2017) have considerably improved resistance compared to for instance Zebra (2001) and Bjarne (2002). The best varieties are far from reaching the resistance levels of well-known resistance sources like Sumai-3 and N894037. The Chinese variety Ning8343 has been used as a resistance source in Norwegian spring wheat breeding, resulting in several breeding lines (512–70, 512–50, 512–21, 512–87, 512–54) with resistance derived from this source (Table 2).

**Table 2:** Mean phenotypic data for selected spring wheat varieties currently on the market in Norway, sources of resistance and susceptible checks in the NMBU spring wheat panel. Please refer to Online Resource 1 for the full phenotypic data on all lines

Entry number	Category	Name	Country	Population	Mean phenotypic data across environments				
					DON (ppm)	FHB (%)	AE (0–9)	PH (cm)	DH (days)
1401	Market variety	Mirakel	Norway	European	5.8	26.2	6.7	95.3	53.8
1403	Market variety	Seniorita	Norway	European	6.6	32.5	6.4	86.4	54.9
1587	Market variety	Caress	Sweden	European	7.1	25.2	6.9	75.9	53.7
1174	Market variety	Krabat	Norway	European	8.8	34.7	5.9	78.9	55.0
1011	Market variety	Zebra	Sweden	European	12.1	45.0	4.9	89.0	53.0
1005	Market variety	Bjarne	Norway	European	13.8	44.3	4.8	73.9	52.3
1627	Resistance source	N894037	China	Others	2.8	16.5	7.4	82.7	55.6
1338	Resistance source	Sumai-3	China	Others	2.9	8.6	6.4	97.2	54.8
1084	Resistance source	512–70	Norway	European	3.0	11.1	5.9	98.0	50.9
1082	Resistance source	512–50	Norway	European	3.4	15.8	7.4	92.3	52.4
1102	Resistance source	Nobeokabouzu	Japan	Others	3.7	14.2	5.7	100.9	52.2
1081	Resistance source	512–21	Norway	European	4.2	16.8	6.2	96.7	51.9
1085	Resistance source	512–87	Norway	European	5.5	18.8	6.1	77.6	51.2
1114	Resistance source	Ning 8343	China	Others	6.6	19.1	6.4	86.3	58.7
1106	Resistance source	Frontana	Brazil	Others	7.0	22.0	3.9	111.3	57.3
1083	Resistance source	512–54	Norway	European	7.8	34.6	5.8	79.9	52.1
1116	Susceptible check—adapted	Vinjett	Sweden	European	15.3	45.4	3.5	86.6	53.2
1634	Susceptible check—exotic	Gamenya	Australia	Others	32.9	56.7	3.7	81.0	52.5

### Linkage disequilibrium and population structure

The estimated *r*^2^ for half decay was 0.22 for the NMBU spring wheat panel and the estimated genome-wide half decay distance was 1 cM. (The markers within the half decay distance of 1 cM were considered to be located in the same QTL on the genetic map.)

**Fig. 2:**
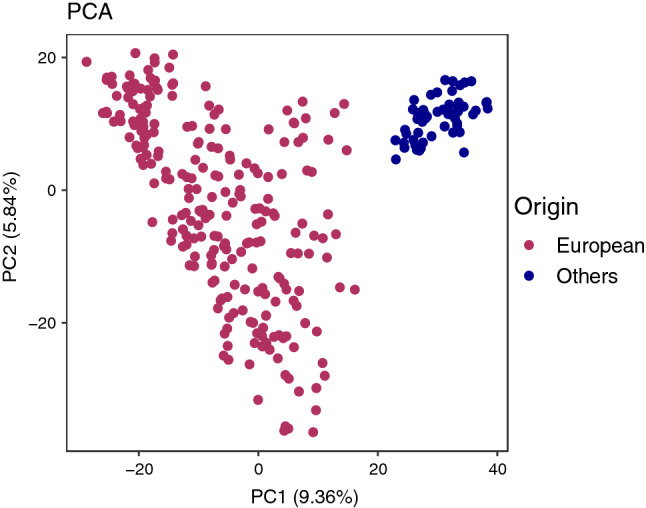
Principal component analysis based on the 25 K data showing the population structure of NMBU spring wheat panel, which is divided mainly into two groups—European and others (lines from outside the Europe such as from CIMMYT, China and USA)

PCA of the marker data confirmed the presence of two groups in the NMBU spring wheat panel, with principal component 1 (PC1) clearly separating the European lines from those originating outside Europe (Fig. 2). For this reason, association analysis was done separately for the complete panel and the European panel.

### Genome-wide association mapping

**Fig. 3:**
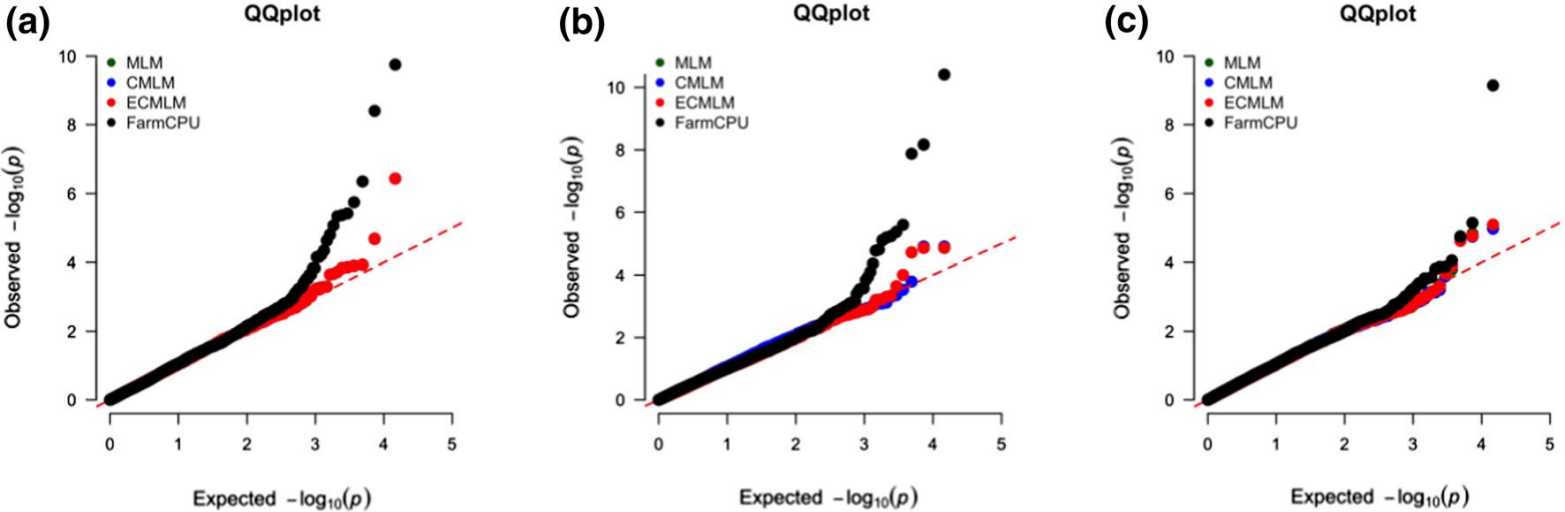
QQ plots of **a** AEmean, **b** DONcPHDHmean and **c** FHBcPHDHmean for different GWAS models—GLM, CMLM, ECMLM and FarmCPU, using the 25 K genotype data

Various GWAS models were tested on the available data, from which FarmCPU was chosen for its proven efficiency over other models in several recent studies (Liu et al. 2016; Kaler et al. 2020). Also, FarmCPU-based QQ plots showed more significant p-values compared to other models (Fig. 3).

Association mapping detected significant marker–trait associations (MTAs) on several chromosomes for each trait. The most important and robust MTAs for the three traits FHBcPHDH, DONcPHDH and AE across the different environments (location by year combinations) amounted to thirteen significant QTL in total. These QTL are located on the following chromosomes (the number following the chromosome number denotes different QTL region on the same chromosome, physical positions in parentheses): *Qfhb.nmbu.1A.1* (9–46 Mbp), *Qfhb.nmbu.1A.2* (520–590 Mbp), *Qfhb.nmbu.3A.1* (683–738 Mbp), *Qfhb.nmbu.3B.1* (7–9.77 Mbp), *Qfhb.nmbu.4A.1* (537–607 Mbp), *Qfhb.nmbu.4A.2* (721–743 Mbp), *Qfhb.nmbu.4B.1* (527–609 Mbp), *Qfhb.nmbu.5A.1* (480–552 Mbp), *Qfhb.nmbu.6A.1* (608–609 Mbp), *Qfhb.nmbu.6B.1* (630–688 Mbp), *Qfhb.nmbu.7A.1* (120–129 Mbp) and *Qfhb.nmbu.7A.2* (670–710 Mbp) (Table 3). All those detected MTAs on specified chromosomes were found using genotyping with 35 and 25 K SNP chips. And most of these were detected in both complete and European panels, except for two QTL regions which were specific only to the complete panel *Qfhb.nmbu.1A.2* (520–590 Mbp) and *Qfhb.nmbu.4A.1* (537–607 Mbp), and one QTL region specific to the European panel on chromosome 4B, QTL *Qfhb.nmbu.4B.1* (527–609 Mbp) (Table 3). In addition, a significant MTA was detected at the *Fhb1* locus on chromosome 3B, *Qfhb.nmbu.3B.1* (7–9 Mbp). This was found significant in two environments, along with the across environment means and PC scores for DONcPHDH. This MTA was only detected in one environment (Tulln, Austria in 2020) for FHBcPHDH and none for AE (Table 3). However, this MTA was only detected in the complete panel but not in the European panel.

**Table 3:** Overview of the most significant and consistent QTL detected in the present GWAS study, and comparison with previously published FHB resistance QTL based on positions on the wheat reference genome assembly (RefSeq v1.0; International Wheat Genome Sequencing Consortium (IWGSC) et al. 2018)

QTL name	Physical map position (Start–End, Mbp)	− log (*p*) value	Trait and number of locations the QTL was detected	NMBU spring wheat panel	Published references
*Qfhb.nmbu.1A.1*	9–46	3.05–13.27	AE (2), FHBcPHDH (3), DONcPHDH (4)	Complete and European panels	Jiang et al. (2007a, b), Buerstmayr et al. (2009), Liu et al. (2009), Sari et al. 2018), Venske et al. (2019) and Zhu et al. (2020)
*Qfhb.nmbu.1A.2*	520–590	3.04–8.16	AE (2), FHBcPHDH (2), DONcPHDH (4)	Complete panel	Venske et al. (2019) and Zheng et al. (2020)
*Qfhb.nmbu.3A.1*	683–738	3.09–12.27	AE (4), FHBcPHDH (3), DONcPHDH (2)	Complete and European panels	Venske et al. (2019)
*Qfhb.nmbu.3B.1*	7–9	3.49–12.86	AE (0), FHBcPHDH (2), DONcPHDH (1)	Complete panel	Anderson et al. (2001), Liu et al. (2006) and Buerstmayr et al. (2009)
*Qfhb.nmbu.4A.1*	537–607	3.20–9.21	AE (1), FHBcPHDH (2), DONcPHDH (4)	Complete panel	Zheng et al, 2020
*Qfhb.nmbu.4A.2*	721–743	3.09–8.40	AE (3), FHBcPHDH (0), DONcPHDH (2)	Complete and European panels	In this present study
*Qfhb.nmbu.4B.1*	527–609	3.02–6.41	AE (0), FHBcPHDH (0), DONcPHDH (2)	European panel	In this present study
*Qfhb.nmbu.5A.1*	480–552	3.0–12.49	AE (4), FHBcPHDH (2), DONcPHDH (2)	Complete and European panels	Venske et al. (2019) and Zheng et al. (2020)
*Qfhb.nmbu.6A.1*	450–531	3.06–6.66	AE (4), FHBcPHDH (1), DONcPHDH (1)	Complete and European panels	In this study
*Qfhb.nmbu.6A.2*	608–609	3.09–5.73	AE (3), FHBcPHDH (4), DONcPHDH (4)	Complete and European panels	Ruan et al. (2020)
*Qfhb.nmbu.6B.1*	630–688	3.03–6.55	AE (4), FHBcPHDH (4), DONcPHDH (4)	Complete and European panels	In this present study
*Qfhb.nmbu.7A.1*	120–129	3.05–3.86	AE (1), FHBcPHDH (3), DONcPHDH (5)	Complete and European panels	In this present study
*Qfhb.nmbu.7A.2*	670–710	3.50–25.45	AE (2), FHBcPHDH (3), DONcPHDH (1)	Complete and European panels	Semagn et al. (2007), Buerstmayr et al. (2009), Sari et al. (2018), Wu et al. (2019) and Ruan et al. (2020)

Since the detected significant MTAs were in the near similar QTL regions (physical positions) for both the genotypic datasets, we will hereafter present further analysis using the 25 K data, as it is the latest data and will be used for further validations on a new set of germplasm in this study. The detailed overview of identified significant MTAs detected with the 25 and 35 K genotype datasets for each trait in all the tested environments, across year means and PC scores are reported in Online Resource 3; Supplementary Tables 6–17.

Graphical representation of QTL locations and Manhattan plots from association mapping are shown in Online Resource 2 (Supplementary Figs. 3, 4, 5, 6 and 7).

### Haplotype analysis

Seven QTL regions were selected for haplotype analysis, and the associated markers in those selected regions were used to construct haplotypes based on the criteria mentioned in the methods section. Marker information of haplotypes and number of haplotypes are shown in Online Resource 3; Supplementary Table 12, and the number of haplotype alleles varied among the selected QTL regions ranging from three to six.

**Fig. 4:**
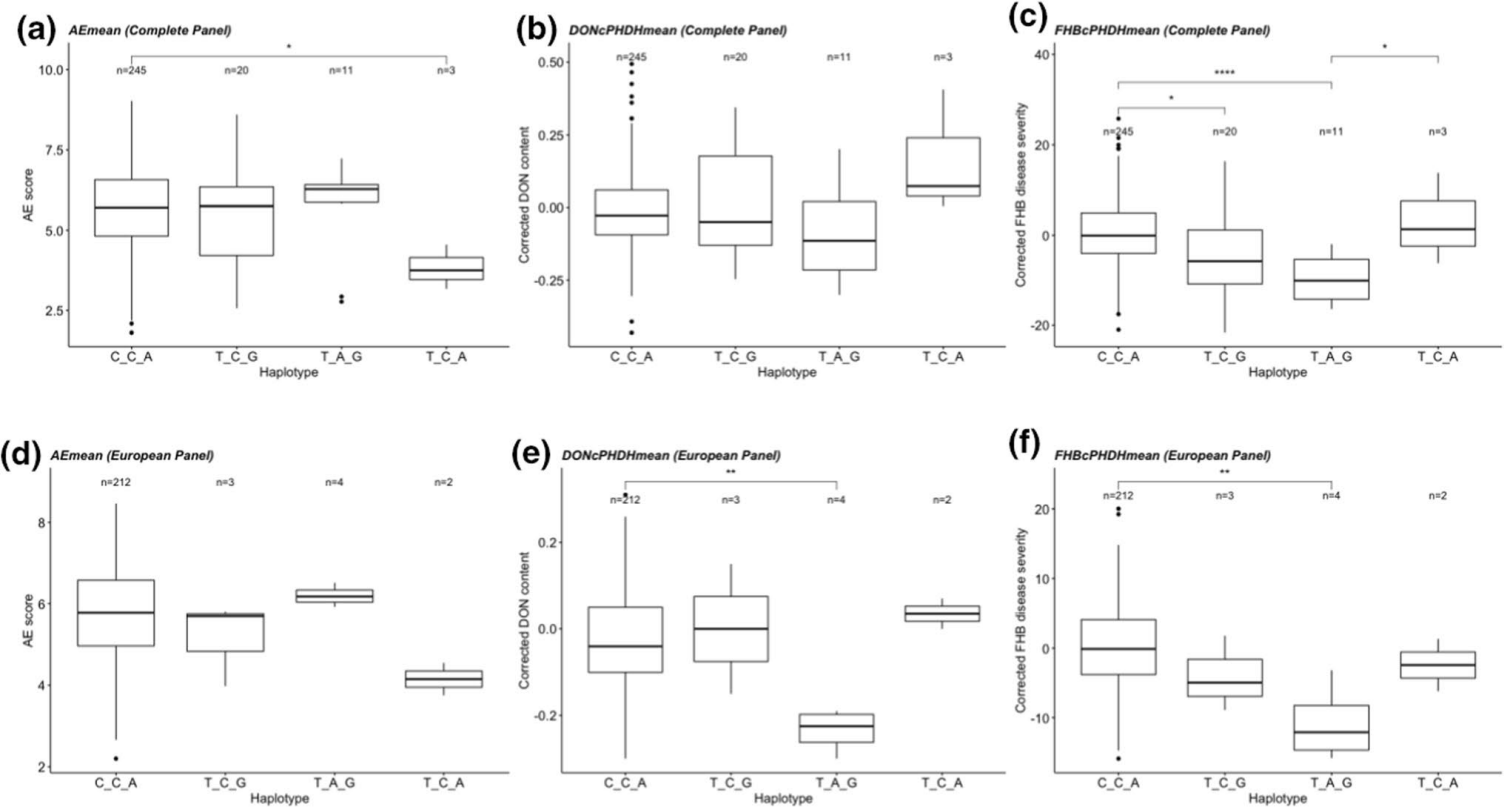
Boxplot showing the haplotype effect of QTL on chromosome 7A based on the **a** mean AE, **b** mean corrected DON content, **c** mean corrected FHB disease severity of complete panel, and **d** mean AE, **e** mean corrected DON content, **f** mean corrected FHB disease severity of European panel. Wilcoxon method is used for pair-wise comparisons (***P < 0.0001, **P < 0.001, *P < 0.05, ns > 0.05). Nonsignificant comparisons are not shown in this figure

For corrected FHB disease severity, only the haplotypes from the QTL regions *Qfhb.nmbu.5A.1* (480–552 Mbp) and *Qfhb.nmbu.7A.2* (670–710 Mbp) showed significant effects on the disease resistance, whereas significant haplotype effects were shown for DON content in all the tested QTL regions. Haplotype effects and significant differences between the haplotypes for *Qfhb.nmbu.7A.2* (670–710 Mbp) are shown in Fig. 4. Haplotype effects of all other tested QTL regions are shown in Online Resource 2 (Supplementary Figs. 8, 9, 10, 11 and 12).

**Fig. 5:**
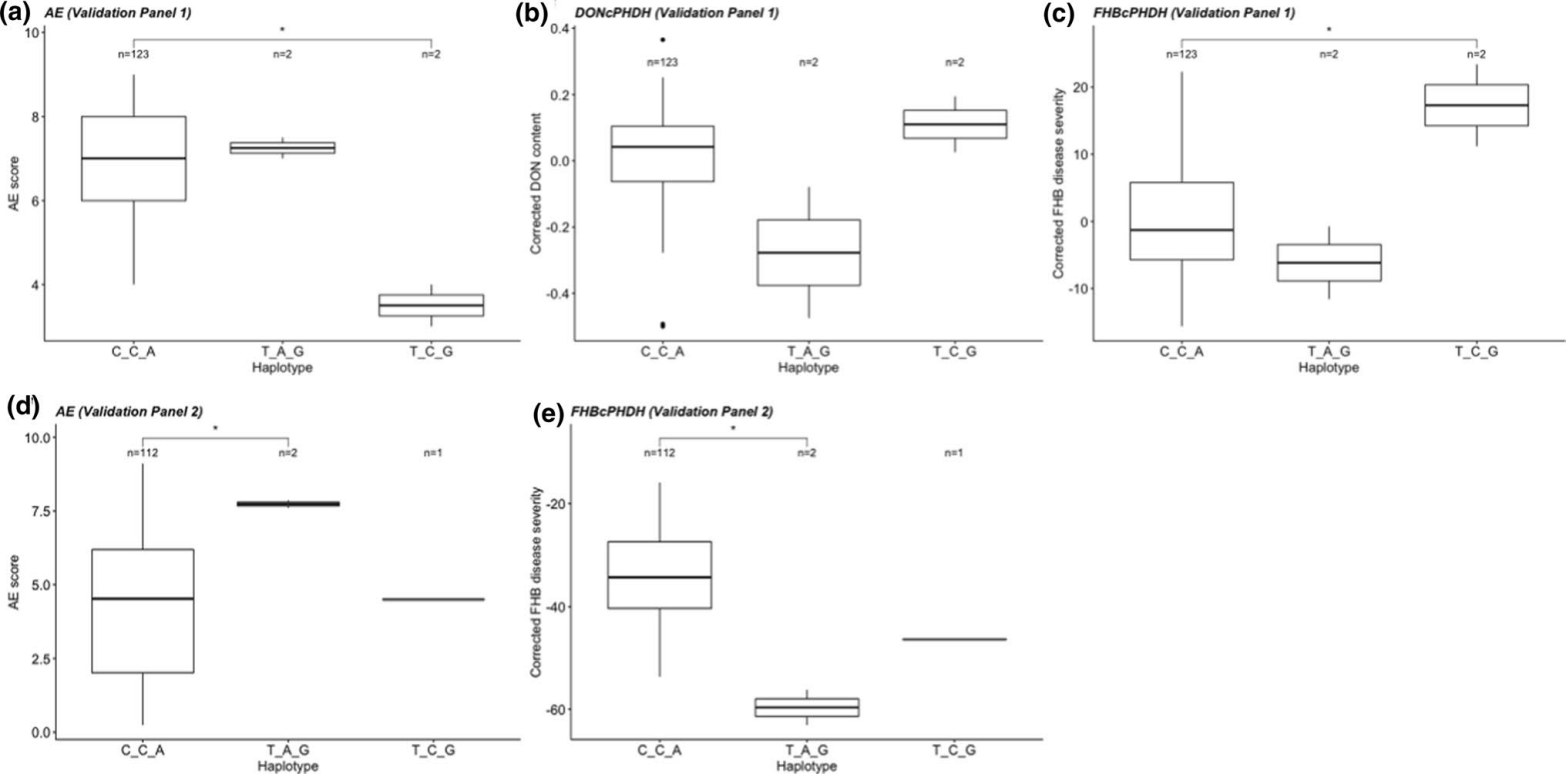
Boxplot showing the haplotype effect of QTL on chromosome 7A based on the **a** AE, **b** corrected DON content, **c** corrected FHB disease severity of validation panel at Vollebekk in 2020 and **d** AE, **e** corrected FHB disease severity of validation panel at Tulln in 2021. Wilcoxon method is used for pair-wise comparisons (***P < 0.0001, **P < 0.001, *P < 0.05, ns > 0.05). Nonsignificant comparisons are not shown in this figure

The same haplotypes from the QTL regions (online Resource 3; Supplementary Table 12) were tested on the validation panel consisting of new breeding lines tested in two different environments (Vollebekk in 2020 and Tulln in 2021). Here, only the QTL region on chromosome 7A (*Qfhb.nmbu.7A.2*) showed significant haplotype effects. For corrected FHB disease severity and AE, the resistant haplotype was significantly different from other haplotypes. A clear trend in the same direction was also shown for DON content in Vollebekk 2020, although not statistically significant due to the low statistical power. Data for DON are not available from the location Tulln, 2021. Only two lines in the validation panel showed the resistant haplotype, and these lines share a similar pedigree with lines having the resistant haplotype on chromosomal region 7A (*Qfhb.nmbu.7A.2*) in the NMBU spring wheat panel (Fig. 5). The most significant SNP markers in this region (*BS00098483_51*, *AX-95248570* and *Kukri_c57593_79*) were converted to breeder-friendly KASP markers and tested on the NMBU spring wheat panel (Online Resource 2; Supplementary Fig. 14). The KASP markers produced similar genotype calls as the original SNP marker data. Primer sequences used for these KASP markers are available in Online Resource 3 (Supplementary Table 14). A few anomalies were noted, which could be caused by using new DNA samples for the KASP genotyping. Nevertheless, the same significant haplotype effects were seen in the KASP validation of complete panel and European panel as compared to the SNP chip data (Online Resource 2; Supplementary Fig. 13).

### Allele stacking

**Fig. 6:**
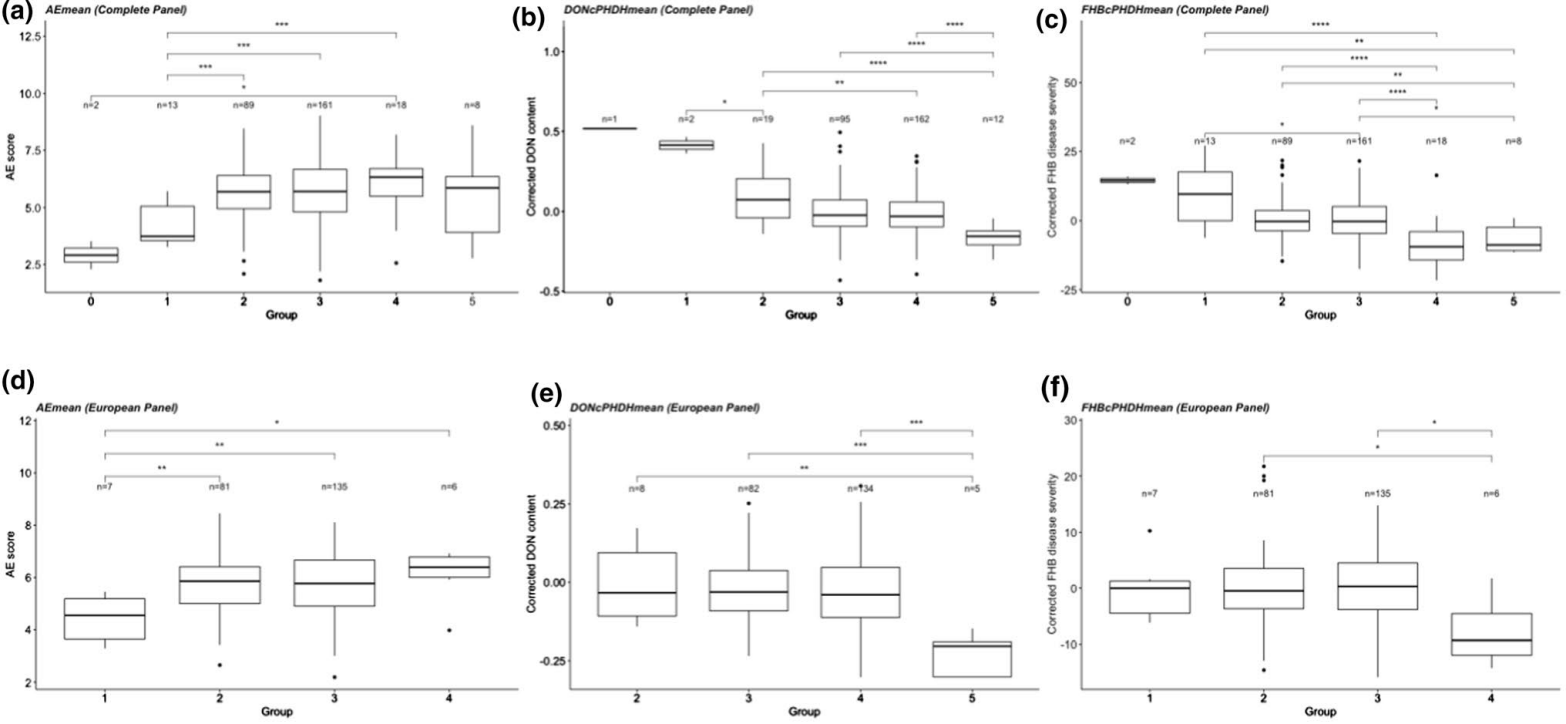
Boxplot showing the effect of number of stacked resistant alleles based on the **a** mean AE, **b** mean corrected DON content, **c** mean corrected FHB disease severity of complete panel, and **d** mean AE, **e** mean corrected DON content, **f** mean corrected FHB disease severity of European panel. Wilcoxon method is used for pair-wise comparisons (***P < 0.0001, **P < 0.001, *P < 0.05, ns > 0.05). Nonsignificant comparisons are not shown in this figure

The effect of stacking different alleles was examined on corrected FHB disease severity, corrected DON content and AE (Fig. 6) in both the complete and the European panels. In the complete panel, the groups ranged from zero to five resistant alleles and the markers selected for the allele stacking are shown in Online Resource 3 (Supplementary Table 13). In the European panel, this number was reduced due to fewer lines and reduced genetic diversity, ranging from one to five resistant alleles. Overall, the lines carrying a greater number of resistant alleles (i.e., four or more) were more resistant than the groups carrying fewer resistant alleles, and significant effects of allele stacking were observed for all three traits (Fig. 6).

## Discussion

Although similar QTL were detected by all the tested models in our study, the FarmCPU model detected fewer QTL than the other models. Since FarmCPU is known to have a better control of false positives and higher power to detect true QTL compared to the other models (Liu et al. 2016; Kaler et al. 2020), we decided to base our analysis on the FarmCPU results. Overall, we detected thirteen significant QTL regions in this study. Five of them were of major importance based on different factors such as consistency across the environments and traits and considering the LD between the significant markers in the respective QTL region. Significant markers from these five QTL regions were tested for their effect on FHB disease resistance traits with the help of haplotype and allele stacking analyses. From these analyses, we were able to identify the resistant haplotypes and the lines contributing the resistance. These results were further validated by performing haplotype analysis on an independent germplasm (i.e., validation panel), which found a QTL region *Qfhb.nmbu.7A.2* (670–710 Mbp) on 7A to be important for improving FHB disease resistance in future breeding efforts.

### Heritability

Heritabilities for FHB and DON were low when compared to AE and were further reduced in the individual analyses of the European panel, which highlights the contribution of genetic diversity for resistance by the exotic lines. The association mapping for FHB resistance revealed several QTL over all chromosomes, but only some of those were consistent in two or more environments. For DON, the disease assessment was performed measuring the content by GC–MS, which is not subjected to the same rate of error as the visual scoring of FHB in the field. The association mapping for DON also revealed several QTL in each environment, and some of the QTL were consistent in two or more environments. These findings suggest that G x E interactions play a significant role in the NMBU spring wheat panel. Therefore, the QTL that were significant in two or more environments for FHB and DON should be emphasized for resistance breeding in the future.

### Trait correlations

DH has been reported to be negatively correlated with FHB (Emrich et al. 2008), while in our study the correlation between DH and FHB was very low and negative and not significant. Correlations between DH and FHB may have confounding effect on the association mapping results, because weather conditions (humidity and temperature) during anthesis can have a huge impact on the success of FHB infection. Hence, days to heading (or anthesis) might affect the disease scores when the germplasm differ in earliness and correlations can be positive or negative, depending on weather conditions in different years. It is therefore important to correct for this factor in individual nurseries. Since we observed a positive and significant correlation between DON and DH, we decided to correct both DON and FHB for DH in the association analyses to avoid any confounding effects of this trait.

Also, PH has been reported to be associated with FHB (Mao et al. 2010; Lu et al. 2013; Kubo et al. 2013). The correlation analyses in the present study further confirm these reports as both FHB and DON were negatively correlated with PH. In wheat production, taller plants may lodge when fertilized and may also make use of modern machinery more difficult, and the preferred plants are therefore the shorter ones. To avoid the confounding effect of PH, FHB and DON were also corrected for PH.

Correlation of AE with FHB and DON was found to be negative and highly significant in this study. High AE has been proposed as a valuable passive resistance trait to avoid FHB infection. Many studies (Skinnes et al. 2010); Lu et al. 2013; Kubo et al. 2013; Buerstmayr and Buerstmayr 2015; He et al. 2016a, b) have reported the correlation of AE with FHB and DON and detected lower FHB infections and DON content in lines with high AE, and suggested high AE to contribute to Type I resistance. These studies clearly suggested that lines with low FHB severity could be achieved by selecting genotypes that displayed high AE but also pointed out that lines that shed anthers well still got infected by FHB. All these findings were further confirmed by Lu et al. (2013), which reported AE to be positively correlated with Type I resistance to FHB in a biparental mapping population and also detected several AE QTL to coincide with FHB QTL. In the association mapping of the present study, we considered only the significant QTL consistent across FHB, DON and AE to be of foremost importance and used them for validation by haplotype analysis. These findings further validate the correlation between AE and FHB, and DON. Therefore, searching for genotypes with high AE could possibly be a valuable contribution to the resistance breeding for FHB. AE is easy to score in the field and shows higher heritability than FHB and DON as demonstrated by the calculated heritabilities in this study. Fusarium resistance breeding can be enhanced by screening lines for high AE before the more laborious and costly FHB testing (Skinnes et al. 2008).

Several studies have reported correlation between FHB and DON in wheat both in segregating material and in collections of varieties with different resistance level (Bai et al. 2001; Miedaner et al. 2003; Snijders 2004; Ji et al. 2015; Hofgaard et al. 2016). The correlation between FHB and DON in the present study was also very significant and positive with a correlation coefficient of 0.66. Reports of the correlation between FHB and DON suggest a complicated relationship, and Bai et al. (2001) performed thus a study with 116 cultivars and breeding lines of wheat to get further insight into this relationship. The results from their study show that cultivars moderately resistant and moderately susceptible to FHB usually had higher DON levels than resistant cultivars, but that there also were exceptions, especially for cultivars with a moderate Type II resistance. In the present study, we assume to have assessed a combination of both Type I and Type II FHB resistance in the field, because the field scoring was performed at a late stage in the development of the plants when both the initial infection (Type I) and spread (Type II) had occurred.

### Comparison with reported or confirmed QTL from previous studies

*Fhb1* is a well-characterized QTL descending from the Asian cultivar Sumai-3 and has been found in numerous QTL studies (Anderson et al. 2001; Liu et al. 2006; Buerstmayr et al. 2009) and is well known for conferring Type II resistance and to a lesser extent Type I resistance (Waldron et al. 1999; Anderson et al. 2001; Buerstmayr et al. 2002, 2003; Cuthbert et al. 2006). Diagnostic markers (*wgrb619_730* and *wgrb619_1450*) obtained from the study by Li et al. (2019) for *Fhb1* were included in the genotypic data at a putative region on chromosome 3B in our study, while the physical position was determined based on reference maps (Liu et al. 2006; Pumphrey et al. 2007). These diagnostic SNP markers showed MTAs with highly significant *p*-values in two tested environments and across years for the trait DONcPHDH, whereas it was only significant in one environment for FHBcPHDH and not detected in any environment for AE. These significant MTAs were only detected in the complete panel but not in the European panel, suggesting that the resistance sources for *Fhb1* were found among the non-European lines. Our study demonstrates that *Fhb1* is effective in providing Type III resistance, which is in line with previous studies (Lemmens et al. 2005; Jiang et al. 2007a; Kluger et al. 2015). Its low allele frequency and lower heritability of FHB compared to DON likely contributed to the lack of consistent detection of an *Fhb1* effect for combined Type I and Type II resistance (FHB visual assessment) in the present study. However, *Fhb1* is an important QTL to be considered for practical resistance breeding.

The *Qfhb.nmbu.1A.1* QTL (Table 3) was located in the same region as the major QTL previously reported by Buerstmayr et al. (2009); Liu et al. (2009); and Venske et al. (2019) at the distal end of 1AS. Other studies have located FHB resistance QTL in a similar region: An FHB severity QTL from the Chinese wheat line CJ9306 was mapped to position 27.2 Mb (Jiang et al. 2007a, b) and a GWAS identified an FHB incidence QTL from Chinese elite germplasm in the same region (Zhu et al. 2020), while another recent study (Sari et al. 2018) identified an important FHB QTL for incidence and severity in the region of 1AS in *T. turgidum* ssp. *carthlicum* cv. Blackbird that agrees well with the 1A.1 QTL found in the present study.

Our study revealed *Qfhb.nmbu.7A.2* (Table 3) as a promising QTL for FHB resistance breeding, which we validated on new breeding lines and developed KASP assays for. The resistance haplotype at this locus explained a significant proportion of both Type I + Type II (FHB severity) and Type III (DON resistance) FHB disease resistance. A similar QTL was reported by Ruan et al. (2020) located at 671 Mb, which showed a large effect on all the examined FHB resistance-related traits. Some previous studies also identified a major QTL for Type II resistance based on point inoculation in the vicinity of the *Qfhb.nmbu.7A.2* region through the physical mapping of the SSR markers *gwm276* and *gwm262* to positions of 642.9 and 681.4 Mb (Semagn et al. 2007; Buerstmayr et al. 2009). Two further studies reported FHB resistance QTL in the same chromosomal area: Wu et al. (2019) identified a QTL affecting DON accumulation in the same region of an elite Chinese germplasm and Sari et al. (2018) reported QTL for FHB severity and incidence in the same region from the durum wheat inbred line DT696.

Additionally, several recent studies identified and reported QTL which are in line with the results from our current study. Venske et al. (2019) published a QTLome meta-analysis of FHB resistance, which reported validated QTL from various studies (Liu et al. 2009; Ruan et al. 2020) that are in the same chromosomal regions such as *Qfhb.nmbu.1A.1*, *Qfhb.nmbu.5A.1* and *Qfhb.nmbu.7A.2* (Table 3). Some other QTL corresponding to our study were tested for prediction of candidate FHB response genes based on transcriptomic and proteomic data by Zheng et al. (2020). They identified and reported seventeen putative candidate genes, which are within the QTL base pair intervals of *Qfhb.nmbu.1A.2*, *Qfhb.nmbu.4A.1* and *Qfhb.nmbu.5A.1* of our study (Table 3).

### Prospects for resistance and genomic breeding of European and Nordic wheat germplasm

Many FHB resistance QTL are derived from Asian sources (Buerstmayr et al. 2009; Steiner et al. 2017). This is one of the reasons why we performed association mapping on two different sets of the wheat panel in the present study, aside from the apparent existence of two subpopulations. This was done to enable the detection of resistance sources within adapted material (European and Nordic lines) that might not appear when performing association analysis on the complete panel. Using this approach, it was also possible to detect QTL in the exotic material that are not yet integrated into the European and Nordic breeding material. These QTL will be interesting for breeders to evaluate and possibly utilize in resistance breeding. Being strongly influenced by the environment, QTL for FHB and DON resistance need to show a consistent effect across several environments to be interesting for breeding and valuable for developing new resistant varieties. Studying the association results for each environment many QTL turned up as significant, but when comparing every environment and their significant QTL, many of these QTL were only significant in one or two environments. Hence, we focused on consistent QTL that were significantly detected in two or more environments and choose two different significance thresholds: one for the across environment LSmeans and one for individual environment data. QTL were regarded as significant in across environment data based on the FDR and examined for their consistency over individual environments with a more lenient significance threshold. By this criterion, it was possible to assess the consistency of QTL effects across environments. It was also observed that only one QTL region was specifically significant in the European panel, while two other QTL regions were specific to the complete panel (Table 3). The QTL that are specific to complete panel should be of importance for improving the resistance in the European and Nordic material because they lack the resistant alleles from these significant QTL regions.

The QTL region (*Qfhb.nmbu.7A.2*) on chromosome 7A has an importance in the contribution of FHB disease resistance, which was evident from haplotype analysis of associated markers in this region in both NMBU spring wheat panel and the validation panel consisting of new breeding lines. The resistant haplotype of this QTL region was significantly more resistant from other haplotypes, showing the lowest corrected FHB disease severity and corrected DON content (Fig. 4). There were five European lines with this resistant haplotype, and they are progenies of the Chinese resistant cultivar Ning 8343. Interestingly, two lines in the validation panel shared the same haplotypes and similar pedigree with the lines that showed the resistant haplotype in NMBU spring wheat panel.

Allele stacking demonstrated lower DON content and higher FHB resistance levels among the lines that carried combinations of multiple favorable alleles from the QTL detected in our study. Most of the lines with a higher number of resistant alleles contributing to disease resistance originated from CIMMYT (Gondo-1 and lines derived from crosses with Catbird) and China (Sumai 3, Ning 8343 and CJ9306). This list also includes some adapted lines from Norway and Sweden, including the lines whose resistance source originated from the Chinese cultivar Ning8343 (Runar//Ning8343/Brakar). This signifies the importance of the exotic resistance sources for contributing FHB resistance. Hence, making use of these resistance sources and fixing the resistant alleles in new breeding lines can improve the disease resistance considerably.

## Conclusion and outlook

We have identified thirteen QTL regions for FHB resistance traits in this GWAS, all of which may have potential for a further use in resistance breeding. Most importantly the five QTL regions on chromosomes 1A, 3A, 5A, 6A and 7A were most consistent among all the detected marker–trait associations based on the criteria discussed above. The QTL *Qfhb.nmbu.7A.2* was additionally validated on an independent set of breeding lines, where lines carrying the resistant haplotype shared a similar pedigree with the resistant lines in the NMBU spring wheat panel. Moreover, breeder-friendly KASP assays were developed and validated for this QTL. The resistance sources and QTL identified in this study will facilitate a further genetic improvement of FHB resistance in Nordic and European wheat germplasm by genomic breeding strategies, which enable an accelerated stacking of multiple resistance alleles to develop new improved cultivars.

## Supplementary Information

**Online Resource 1.** Phenotypic data and genotypic data used in the study. Phenotypic data—Information of NMBU spring wheat panel including genotype ID, names, country of origin and the raw and corrected data for each genotype in various environments for each trait for NMBU spring wheat panel and Validation panel. Genotypic data—35 K and 25 K data for 14713 and 21653 SNPs, respectively, of 296 lines for NMBU spring wheat panel including SNP ID, chromosome, physical position and 25 K data for 19679 SNPs of 131 lines of the validation panel, including SNP ID, chromosome, physical position.

**Online Resource 2.** Supplementary figures including the frequency distributions of all the traits tested in different environments, correlations between the traits, Manhattan plots of GWAS with supporting QQ plots, boxplots showing the haplotype effects of QTL validation, KASP validation and cluster plots of KASP markers.

**Online Resource 3.** Supplementary tables containing the information of MTAs including marker name, chromosome number, physical position, p-value, -log10(p-value), FDR-adjusted value, starting position and ending position on chromosomes in bp, information of selected significant markers used for allele stacking and haplotype analysis and information about KASP marker primer sequences.

Below is the link to the electronic supplementary material.

Supplementary file1 (XLSX 45794 kb)

Supplementary file2 (PDF 14478 kb)

Supplementary file3 (XLSX 118 kb)

## Data Availability

All the data from this study, i.e., both phenotypic and genotypic datasets, are included with manuscript submission and other relevant information.

## Abbreviations

AE: Anther extrusion
AR: Anther retention
DH: Days to heading
DA: Days to anthesis
DON: Deoxynivalenol
DONcPHDH: DON corrected for plant height and days to heading
FHB: Fusarium head blight
FHBcPHDH: FHB disease severity corrected for plant height and days to heading
Mbp: Mega base pairs
MTA: Marker–Trait associations
PCA: Principal component analysis
PH: Plant height
tDON: Transformed DON content

## Code availability

The scripts and software used for data analyses in this study are available upon request.
