# Supplementary material for: Genetic architecture of fusarium head blight disease resistance and associated traits in Nordic spring wheat
Source: Theor Appl Genet. 2022 May 21;135(7):2247–63. doi: 10.1007/s00122-022-04109-9 (PMC9271104; doi:10.1007/s00122-022-04109-9)
Supplement: Supplementary file 2 — Supplementary file2 (PDF 14478 kb) [file 122_2022_4109_MOESM2_ESM.pdf]

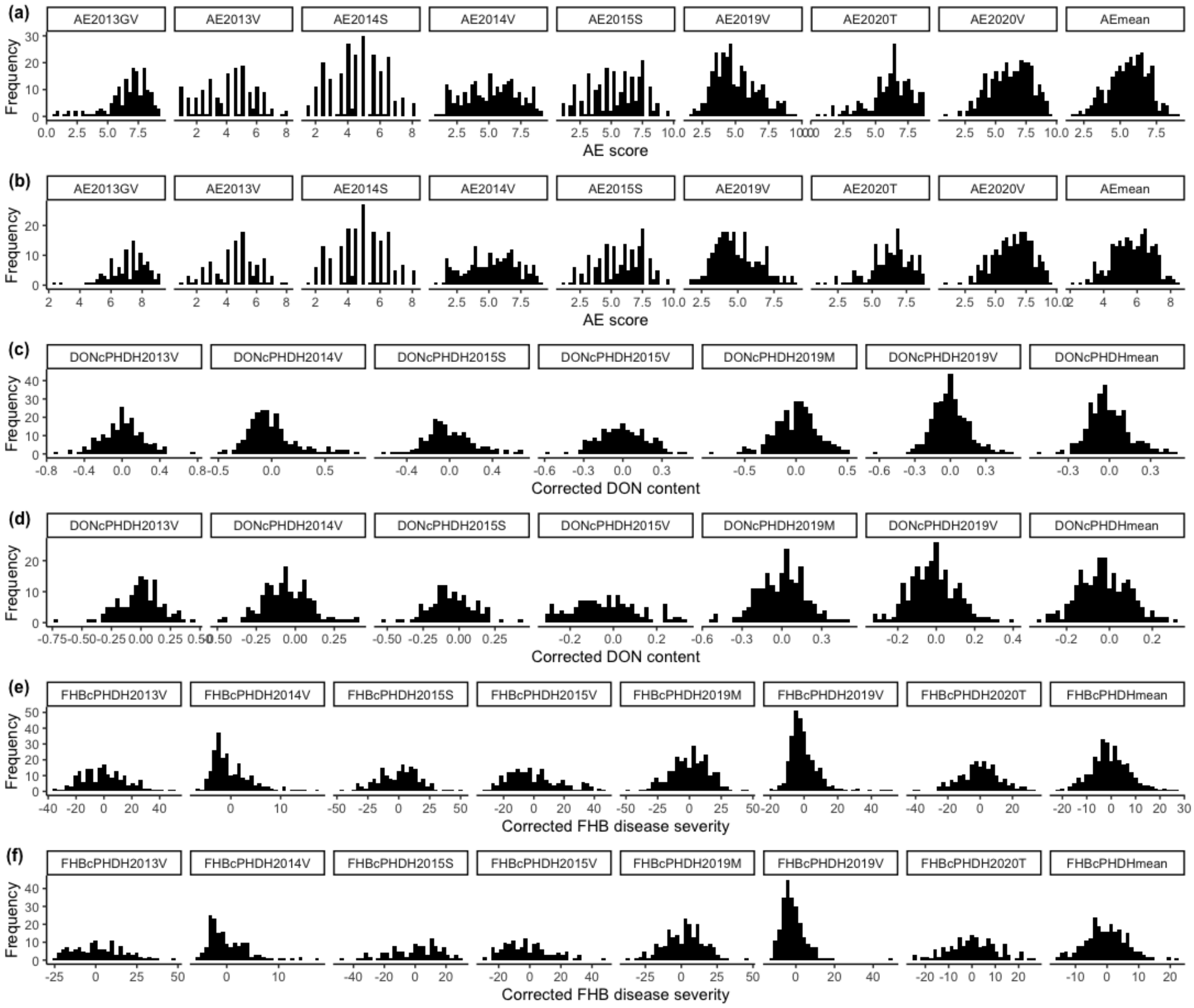

Supplementary figure 1: Histograms of distributions of (a) complete panel, (b) European panel for AE, (c) complete panel, (d) European panel for DONcPHDH and (e) complete panel, (f) European panel for FHBcPHDH for all tested environments and across environment means.

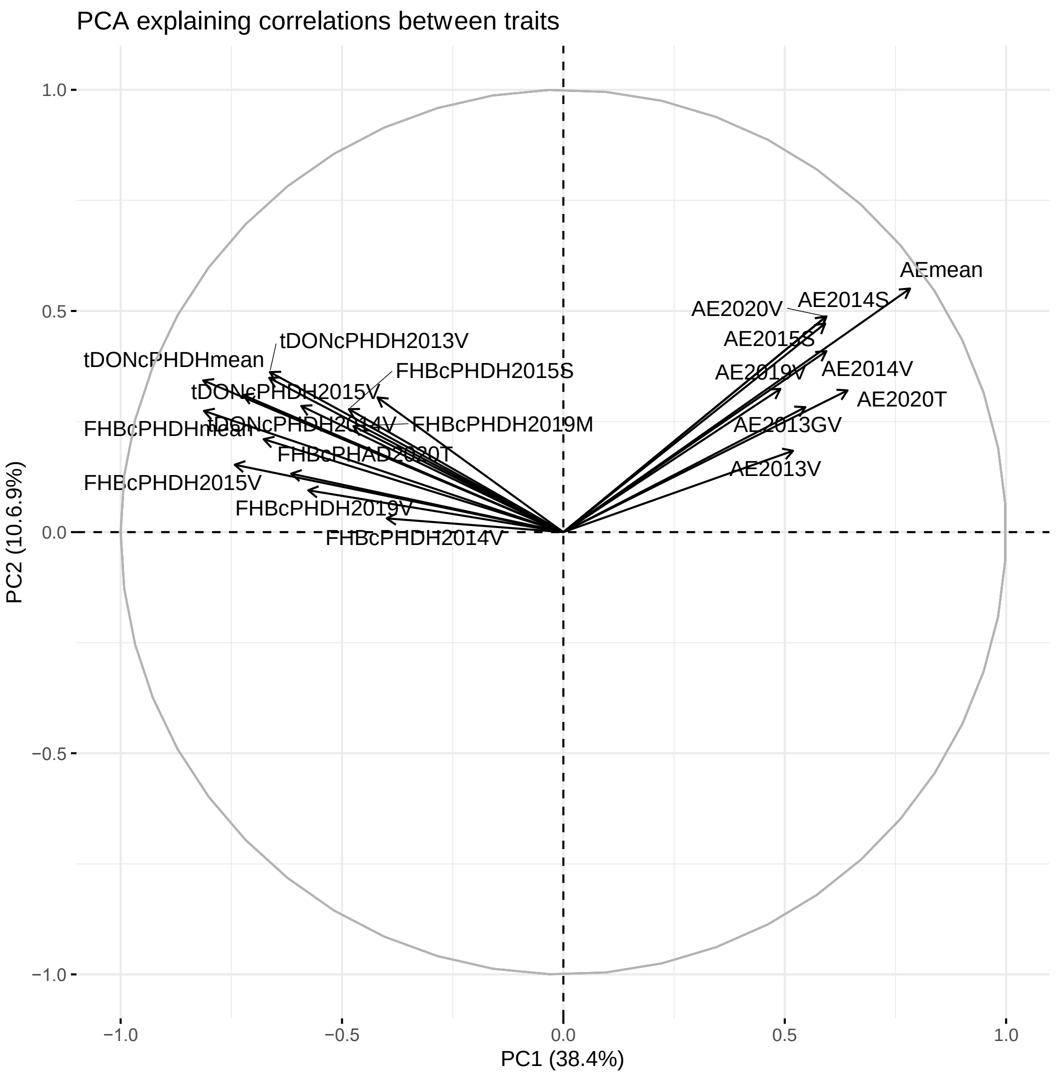

Supplementary figure 2: Principal Component Analysis showing the correlations among the traits AE, DONcPHDH and FHBcPHDH in different tested environments and across environment means.

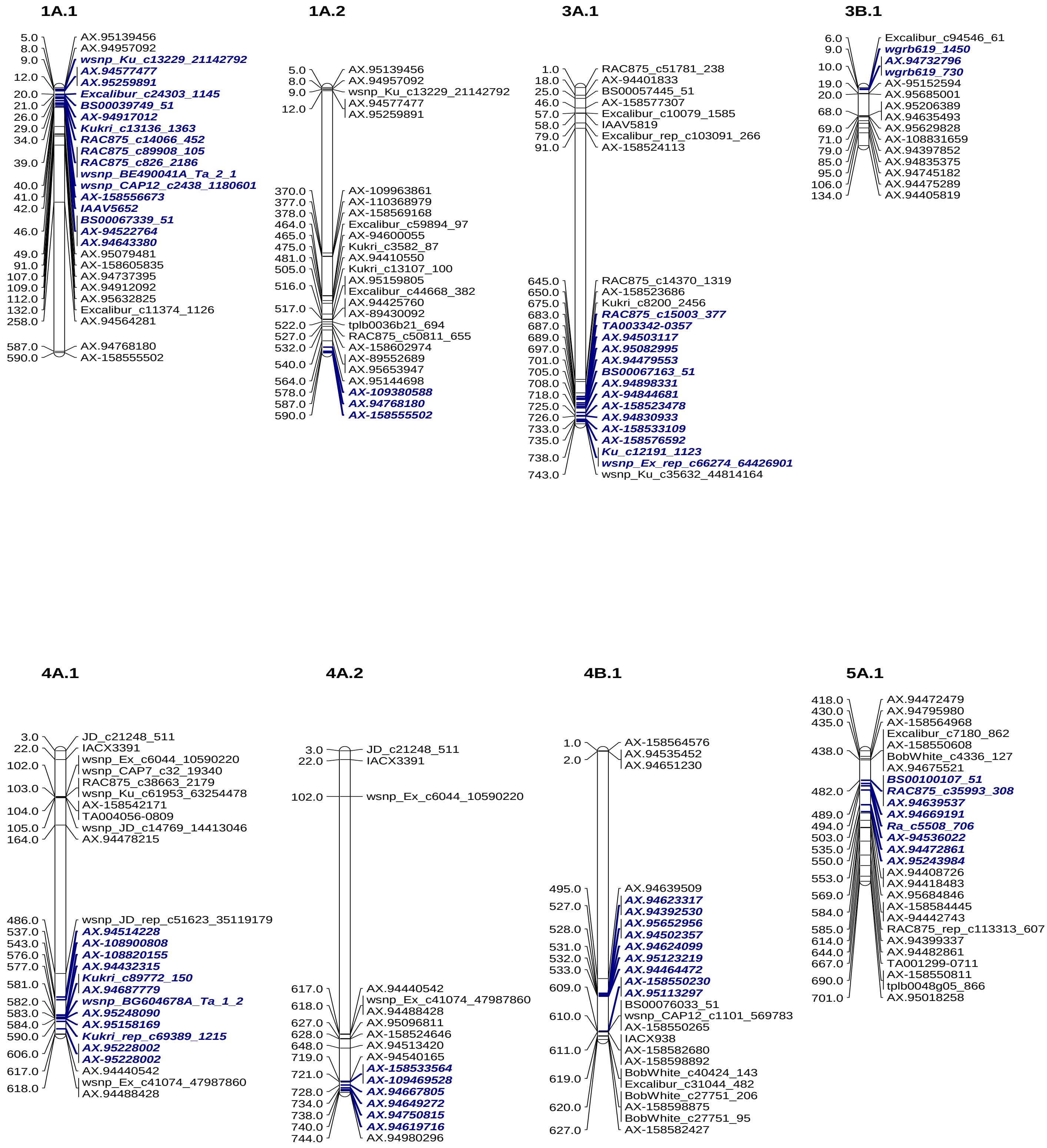

1A.1
5.0 AX.95139456
8.0 AX.94957092
9.0 wsnp_Ku_c13229_21142792
12.0 AX.94577477
AX.95259891
20.0 Excalibur_c24303_1145
21.0 BS00039749_51
26.0 AX-94917012
29.0 Kukri_c13136_1363
34.0 RAC875_c14066_452
RAC875_c89908_105
39.0 RAC875_c826_2186
wsnp_BE490041A_Ta_2_1
40.0 wsnp_CAP12_c2438_1180601
41.0 AX-158556673
42.0 IAAV5652
BS00067339_51
46.0 AX-94522764
AX.94643380
49.0 AX.95079481
91.0 AX-158605835
107.0 AX.94737395
109.0 AX.94912092
112.0 AX.95632825
132.0 Excalibur_c11374_1126
258.0 AX.94564281
587.0 AX.94768180
590.0 AX-158555502
1A.2
5.0 AX.95139456
8.0 AX.94957092
9.0 wsnp_Ku_c13229_21142792
12.0 AX.94577477
AX.95259891
370.0 AX-109963861
377.0 AX-110368979
378.0 AX-158569168
464.0 Excalibur_c59894_97
465.0 AX-94600055
475.0 Kukri_c3582_87
481.0 AX.94410550
505.0 Kukri_c13107_100
516.0 AX.95159805
Excalibur_c44668_382
517.0 AX.94425760
AX-89430092
522.0 tplb0036b21_694
527.0 RAC875_c50811_655
532.0 AX-158602974
540.0 AX-89552689
AX.95653947
564.0 AX.95144698
578.0 AX-109380588
587.0 AX.94768180
590.0 AX-158555502
3A.1
1.0 RAC875_c51781_238
18.0 AX-94401833
25.0 BS00057445_51
46.0 AX-158577307
57.0 Excalibur_c10079_1585
58.0 IAAV5819
79.0 Excalibur_rep_c103091_266
91.0 AX-158524113
645.0 RAC875_c14370_1319
650.0 AX-158523686
675.0 Kukri_c8200_2456
683.0 RAC875_c15003_377
687.0 TA003342-0357
689.0 AX.94503117
697.0 AX.95082995
701.0 AX.94479553
705.0 BS00067163_51
708.0 AX.94898331
718.0 AX-94844681
725.0 AX-158523478
726.0 AX.94830933
733.0 AX-158533109
735.0 AX-158576592
738.0 Ku_c12191_1123
wsnp_Ex_rep_c66274_64426901
743.0 wsnp_Ku_c35632_44814164
3B.1
6.0 Excalibur_c94546_61
9.0 wgrb619_1450
10.0 AX.94732796
wgrb619_730
19.0 AX-95152594
20.0 AX.95685001
68.0 AX.95206389
AX.94635493
69.0 AX.95629828
71.0 AX-108831659
79.0 AX.94397852
85.0 AX.94835375
95.0 AX.94745182
106.0 AX.94475289
134.0 AX.94405819
4A.1
3.0 JD_c21248_511
22.0 IACX3391
102.0 wsnp_Ex_c6044_10590220
wsnp_CAP7_c32_19340
103.0 RAC875_c38663_2179
wsnp_Ku_c61953_63254478
104.0 AX-158542171
TA004056-0809
105.0 wsnp_JD_c14769_14413046
164.0 AX.94478215
486.0 wsnp_JD_rep_c51623_35119179
537.0 AX.94514228
543.0 AX-108900808
576.0 AX-108820155
577.0 AX.94432315
581.0 Kukri_c89772_150
AX.94687779
582.0 wsnp_BG604678A_Ta_1_2
583.0 AX.95248090
584.0 AX.95158169
590.0 Kukri_rep_c69389_1215
606.0 AX.95228002
AX-95228002
617.0 AX.94440542
618.0 wsnp_Ex_c41074_47987860
AX.94488428
4A.2
3.0 JD_c21248_511
22.0 IACX3391
102.0 wsnp_Ex_c6044_10590220
617.0 AX.94440542
618.0 wsnp_Ex_c41074_47987860
AX.94488428
627.0 AX.95096811
628.0 AX-158524646
648.0 AX.94513420
719.0 AX-94540165
721.0 AX-158533564
AX-109469528
728.0 AX.94667805
734.0 AX.94649272
738.0 AX.94750815
740.0 AX.94619716
744.0 AX.94980296
4B.1
1.0 AX-158564576
2.0 AX.94535452
AX.94651230
495.0 AX.94639509
527.0 AX.94623317
AX.94392530
528.0 AX.95652956
AX.94502357
531.0 AX.94624099
532.0 AX.95123219
533.0 AX.94464472
609.0 AX-158550230
AX.95113297
BS00076033_51
610.0 wsnp_CAP12_c1101_569783
AX-158550265
IACX938
611.0 AX-158582680
AX-158598892
619.0 BobWhite_c40424_143
Excalibur_c31044_482
620.0 BobWhite_c27751_206
AX-158598875
BobWhite_c27751_95
627.0 AX-158582427
5A.1
418.0 AX.94472479
430.0 AX.94795980
435.0 AX-158564968
Excalibur_c7180_862
438.0 AX-158550608
BobWhite_c4336_127
AX.94675521
482.0 BS00100107_51
RAC875_c35993_308
AX.94639537
489.0 AX.94669191
494.0 Ra_c5508_706
503.0 AX-94536022
535.0 AX.94472861
550.0 AX.95243984
553.0 AX.94408726
AX.94418483
569.0 AX.95684846
584.0 AX-158584445
AX-94442743
585.0 RAC875_rep_c113313_607
614.0 AX.94399337
644.0 AX.94482861
667.0 TA001299-0711
690.0 AX-158550811
tplb0048g05_866
701.0 AX.95018258

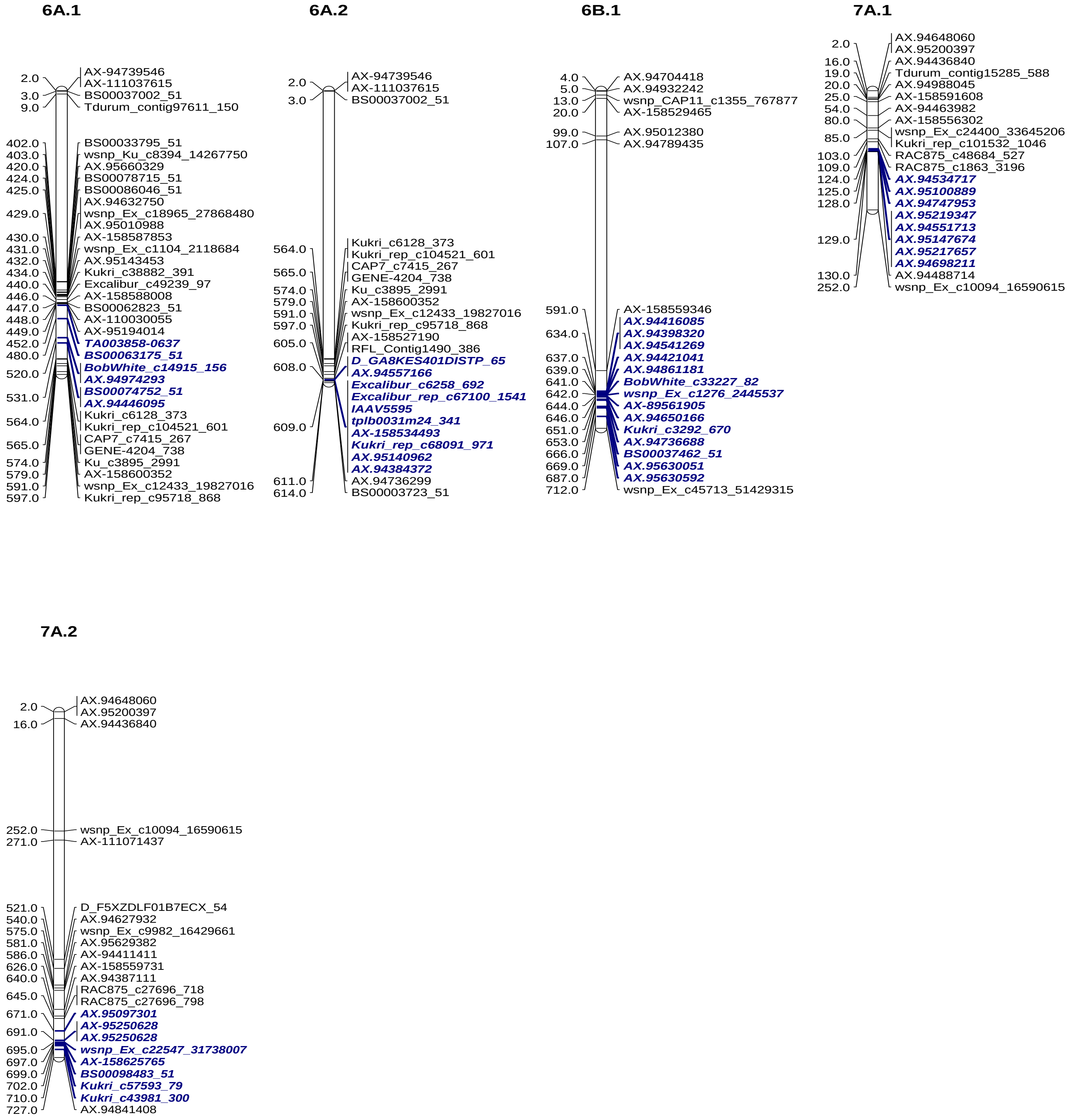

Supplementary figure 3: Chromosome locations of quantitative trait loci (QTL) for FHB resistance that were detected in one or more environments. The positions of marker loci in Mega base pairs (Mbp) are shown along the left side of the linkage groups. Markers are shown on the right of the linkage groups. The most consistently detected markers for each QTL region are highlighted in both italics and blue colour (Note: Only the significant markers of the respective QTL regions from the GWAS results were shown in the maps).

(a)

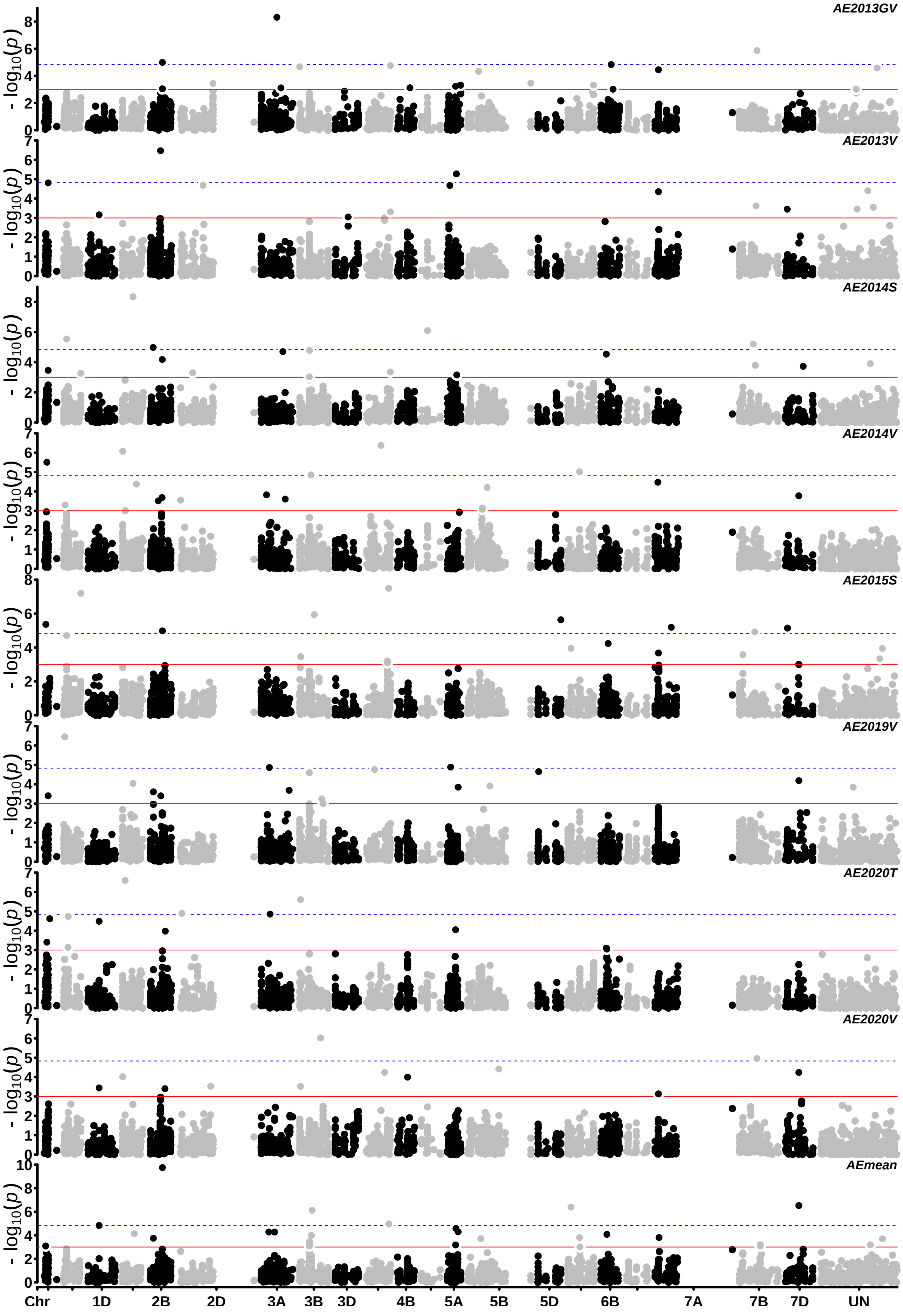
AE2013GV
AE2013V
AE2014S
AE2014V
AE2015S
AE2019V
AE2020T
AE2020V
AEmean
- log10(p)
Chr
1D
2B
2D
3A
3B
3D
4B
5A
5B
5D
6B
7A
7B
7D
UN

(b)

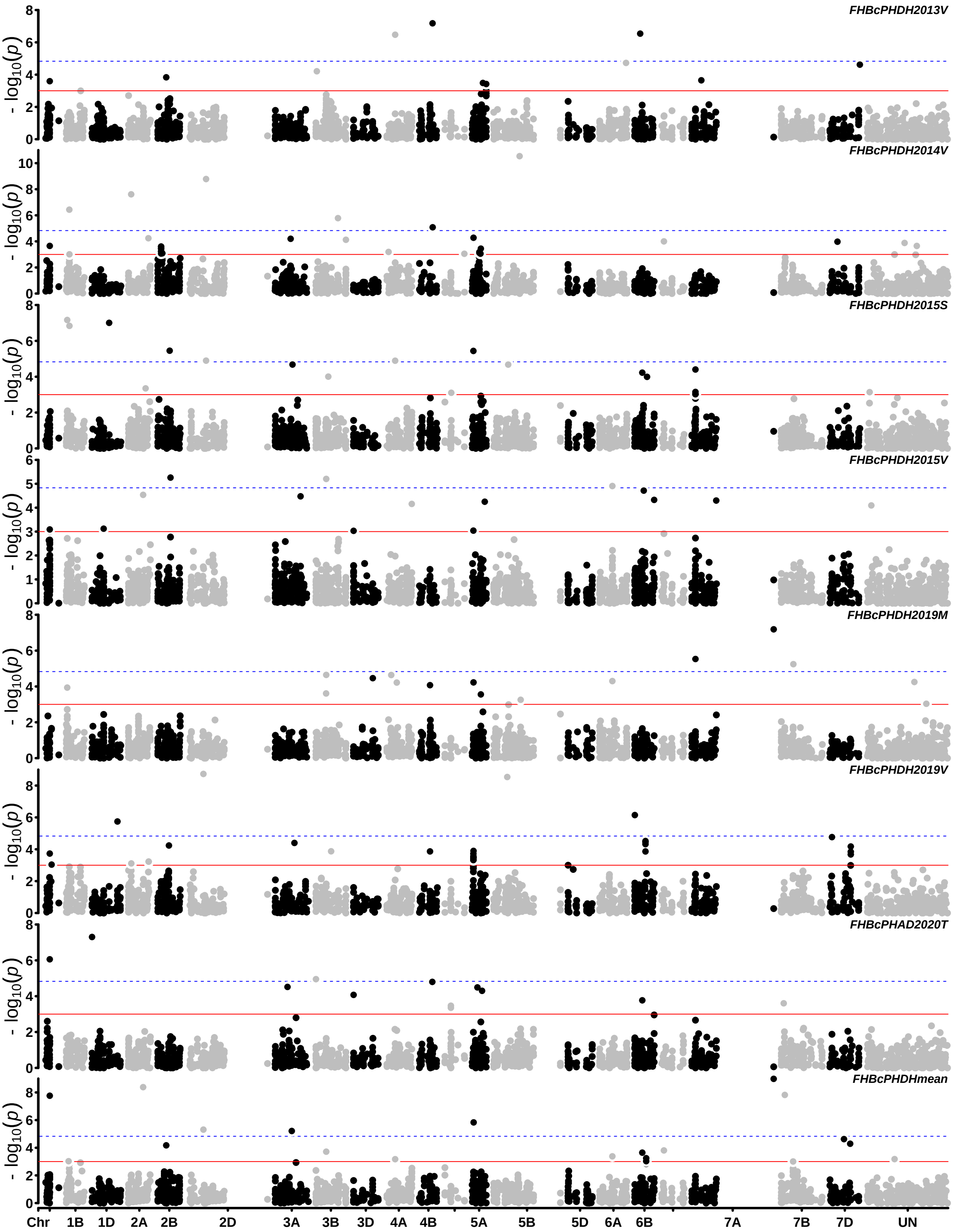
FHBcPHDH2013V
FHBcPHDH2014V
FHBcPHDH2015S
FHBcPHDH2015V
FHBcPHDH2019M
FHBcPHDH2019V
FHBcPHAD2020T
FHBcPHDHmean
- log10(p)
Chr
1B
1D
2A
2B
2D
3A
3B
3D
4A
4B
5A
5B
5D
6A
6B
7A
7B
7D
UN

(c)

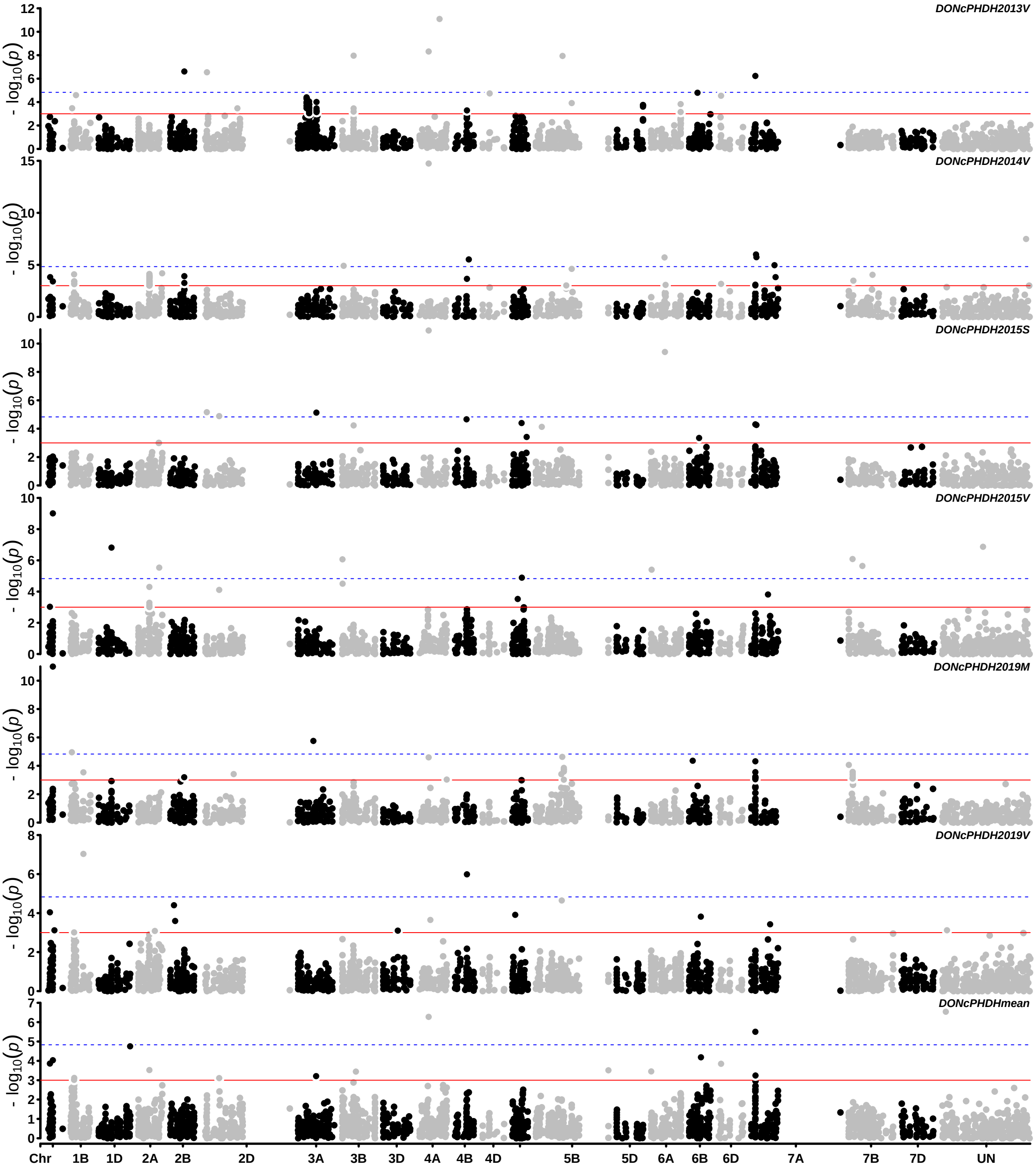
DONcPHDH2013V
DONcPHDH2014V
DONcPHDH2015S
DONcPHDH2015V
DONcPHDH2019M
DONcPHDH2019V
DONcPHDHmean
- log10(p)
Chr 1B 1D 2A 2B 2D 3A 3B 3D 4A 4B 4D 5B 5D 6A 6B 6D 7A 7B 7D UN

(d)

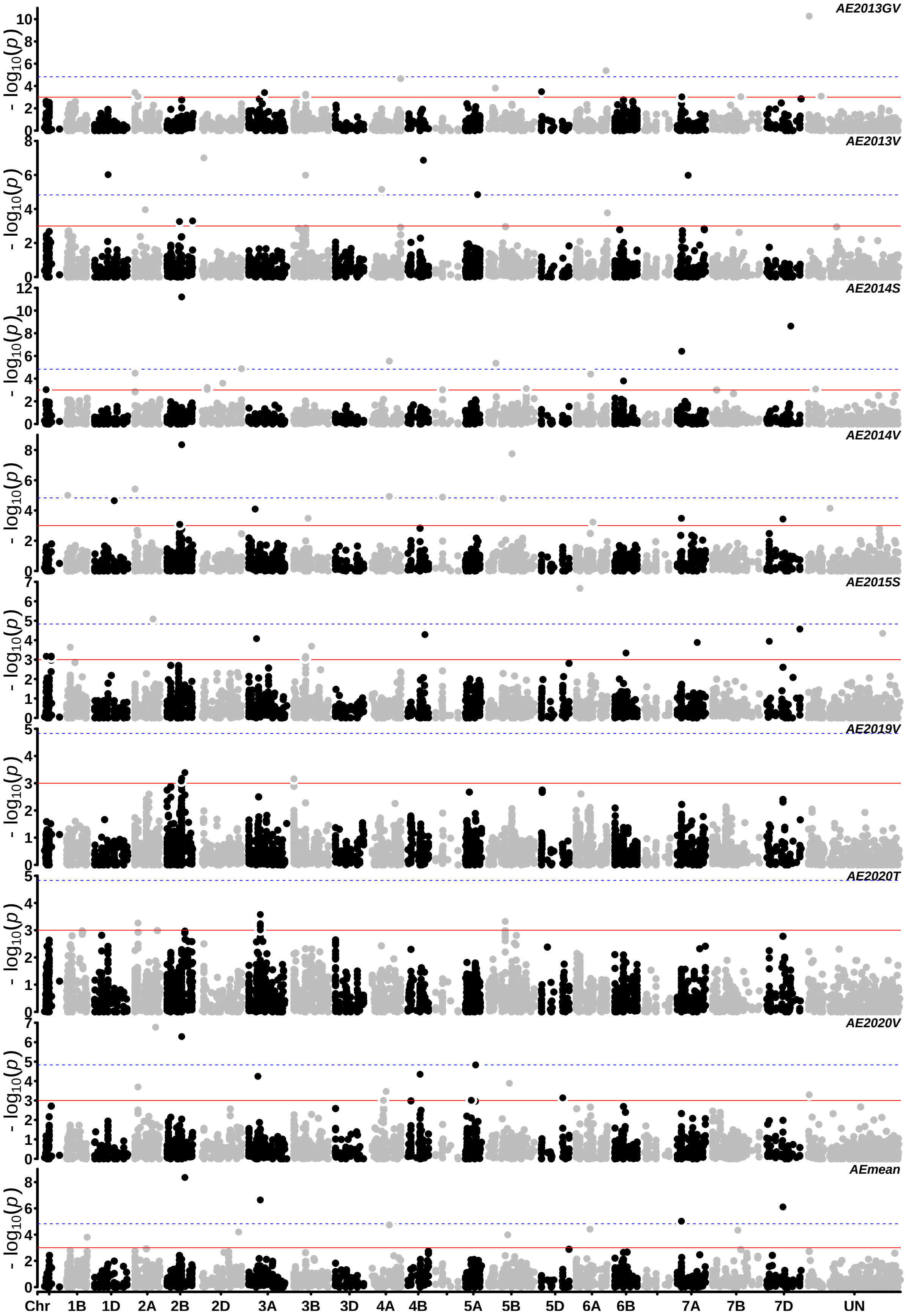
AE2013GV
AE2013V
AE2014S
AE2014V
AE2015S
AE2019V
AE2020T
AE2020V
AEmean
- log10(p)
Chr 1B 1D 2A 2B 2D 3A 3B 3D 4A 4B 5A 5B 5D 6A 6B 7A 7B 7D UN

(e)

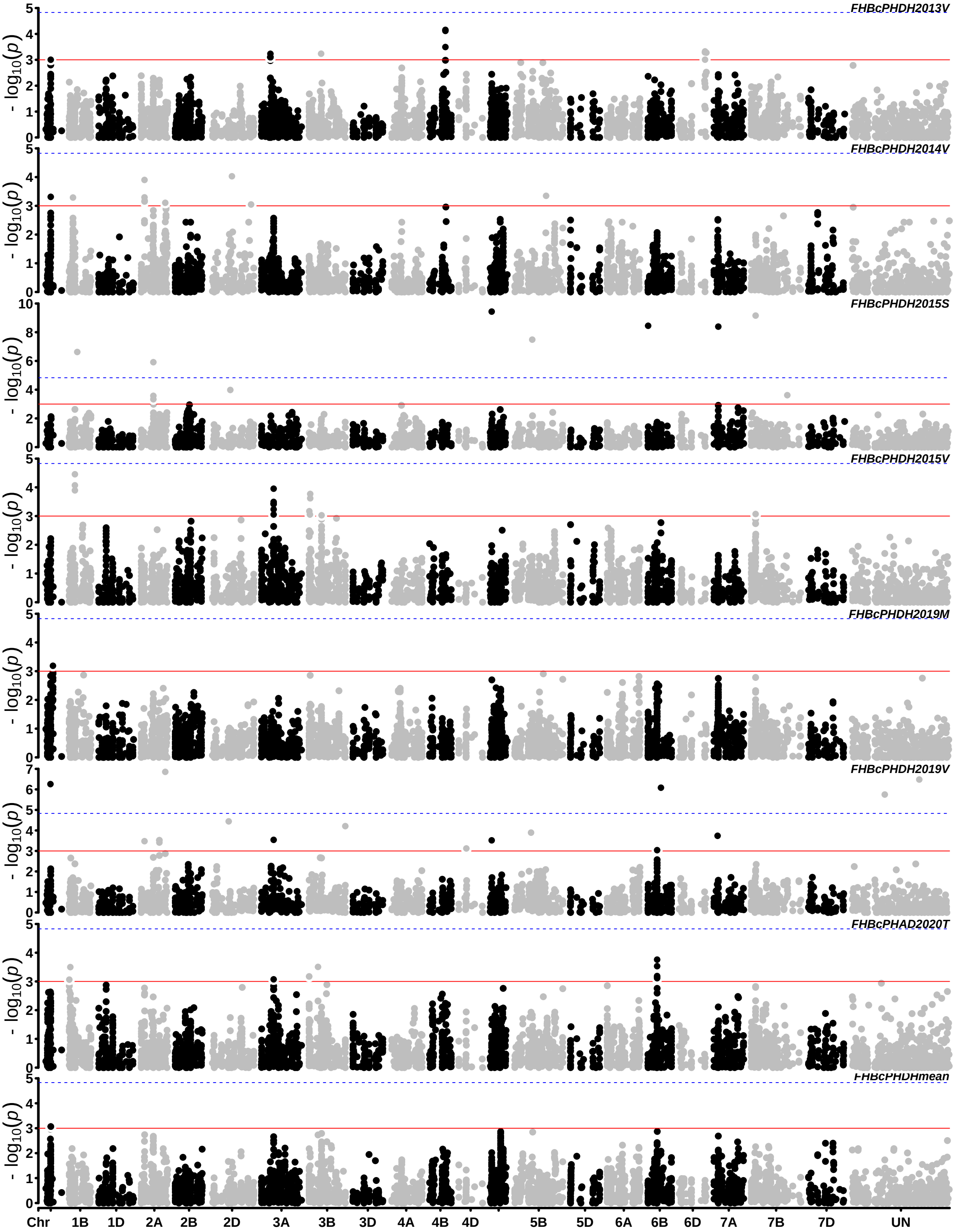
FHBcPHDH2013V
FHBcPHDH2014V
FHBcPHDH2015S
FHBcPHDH2015V
FHBcPHDH2019M
FHBcPHDH2019V
FHBcPHAD2020T
FHBcPHDHmean
- log10(p)
Chr 1B 1D 2A 2B 2D 3A 3B 3D 4A 4B 4D 5B 5D 6A 6B 6D 7A 7B 7D UN

(f)

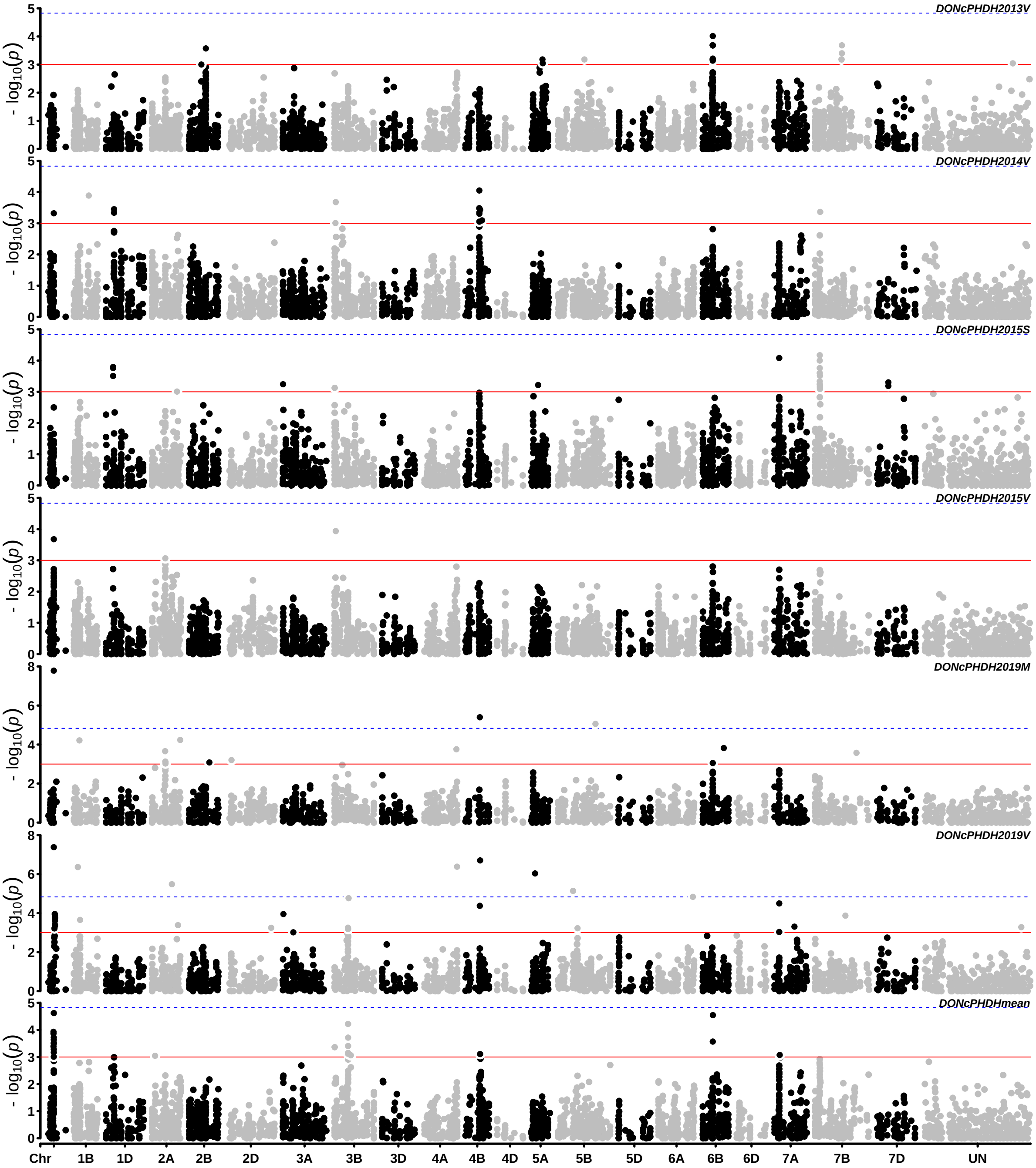

Supplementary figure 4: Manhattan plots of traits AE (a and d), FHBcPHDH (b and e), and DONcPHDH (c and f) in complete panel and European panel respectively for 35K, red line represents significant threshold (-log10p = 3.0) and blue dotted line is FDR @ 0.05% based on across environment means. Suffix to the trait names indicate the names of experimental locations: “GV” for “GreenhouseVollebekk”, “M” for “Morden”, “S” for Staur, “T” for Tulln, and “V” for Vollebekk.

(a)

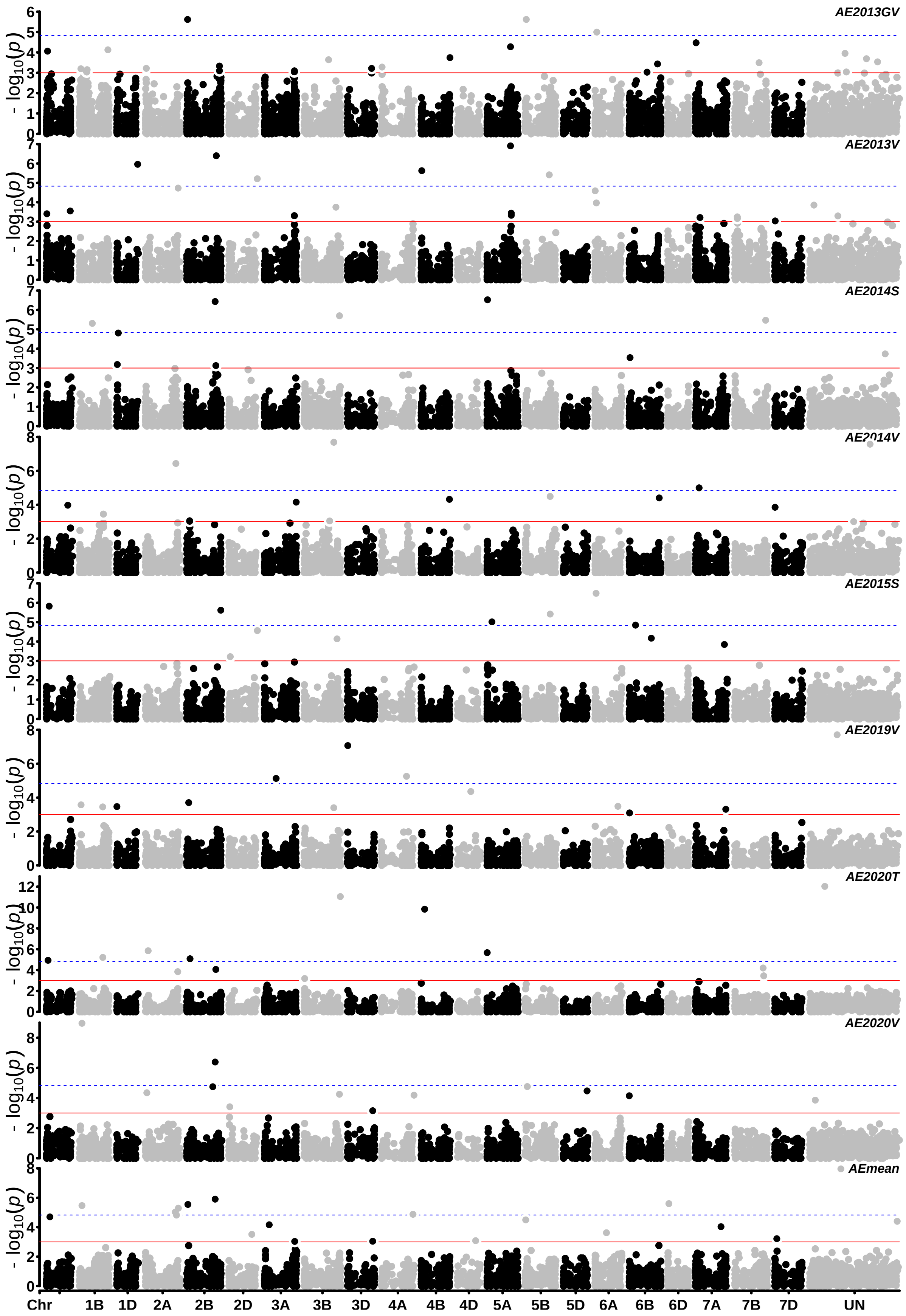
AE2013GV
AE2013V
AE2014S
AE2014V
AE2015S
AE2019V
AE2020T
AE2020V
AEmean
- log10(p)
Chr
1B
1D
2A
2B
2D
3A
3B
3D
4A
4B
4D
5A
5B
5D
6A
6B
6D
7A
7B
7D
UN

(b)

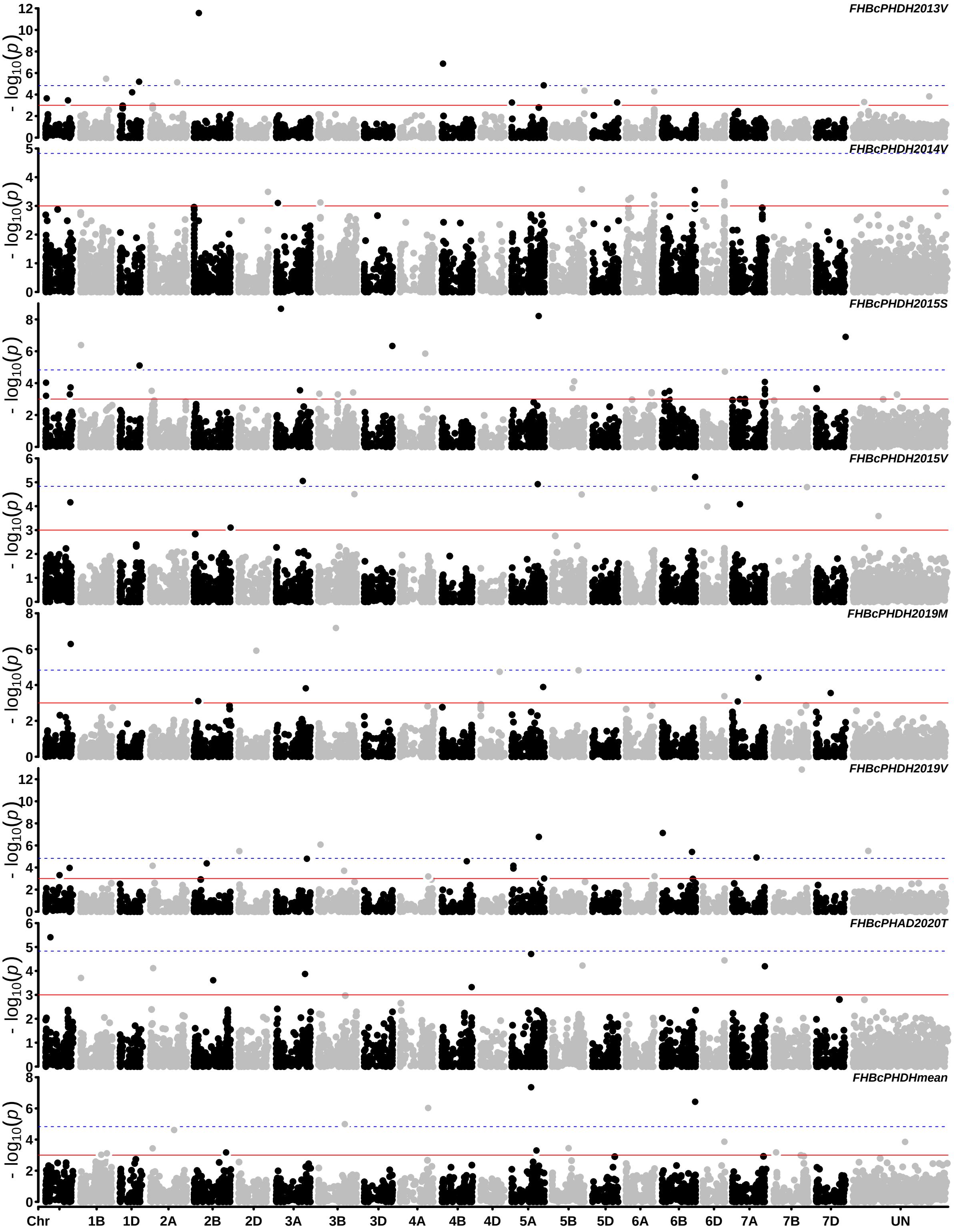
FHBcPHDH2013V
FHBcPHDH2014V
FHBcPHDH2015S
FHBcPHDH2015V
FHBcPHDH2019M
FHBcPHDH2019V
FHBcPHAD2020T
FHBcPHDHmean
$-\log_{10}(p)$
Chr
1B
1D
2A
2B
2D
3A
3B
3D
4A
4B
4D
5A
5B
5D
6A
6B
6D
7A
7B
7D
UN

(c)

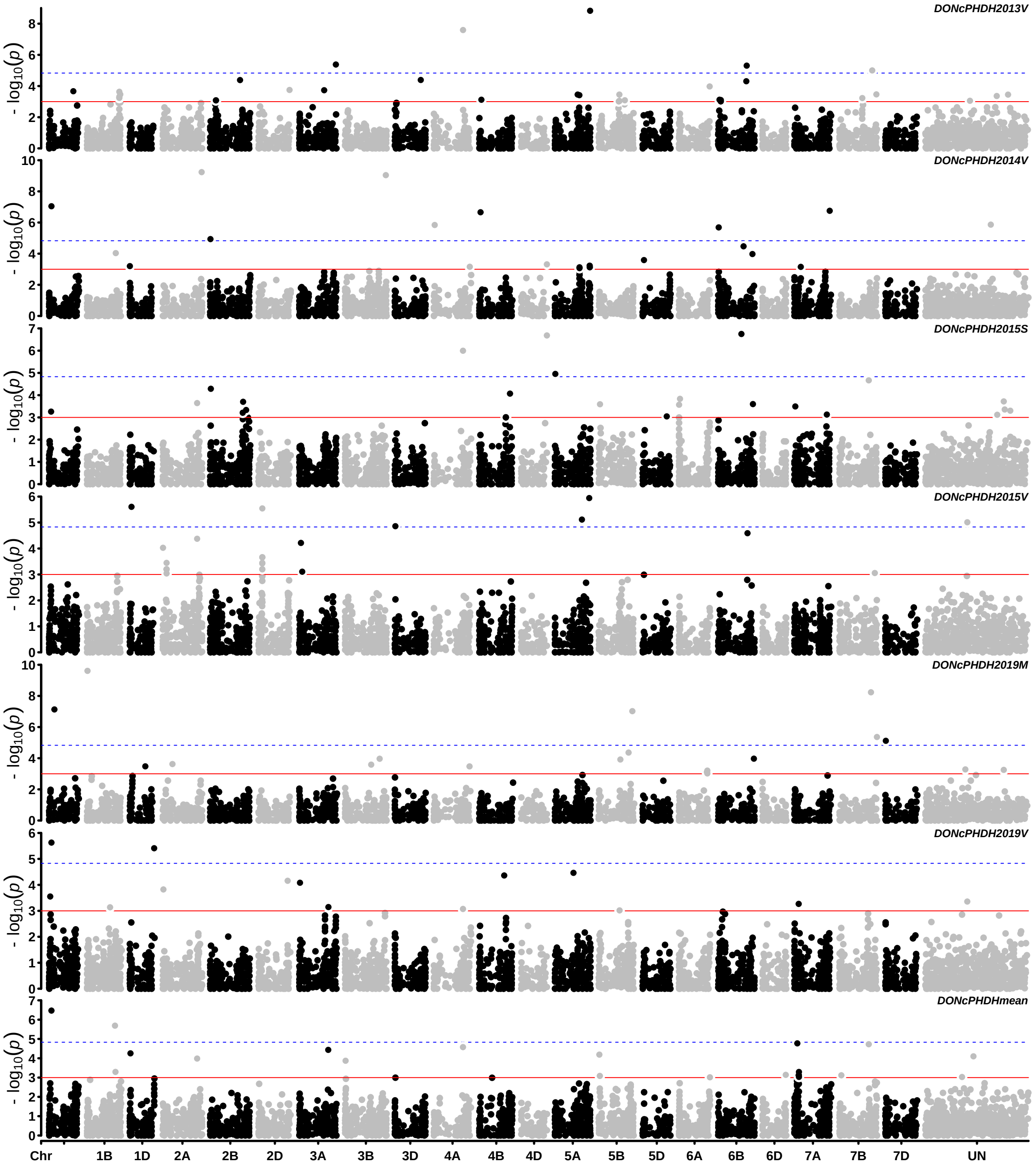
DONcPHDH2013V
DONcPHDH2014V
DONcPHDH2015S
DONcPHDH2015V
DONcPHDH2019M
DONcPHDH2019V
DONcPHDHmean
- log10(p)
Chr 1B 1D 2A 2B 2D 3A 3B 3D 4A 4B 4D 5A 5B 5D 6A 6B 6D 7A 7B 7D UN

(d)

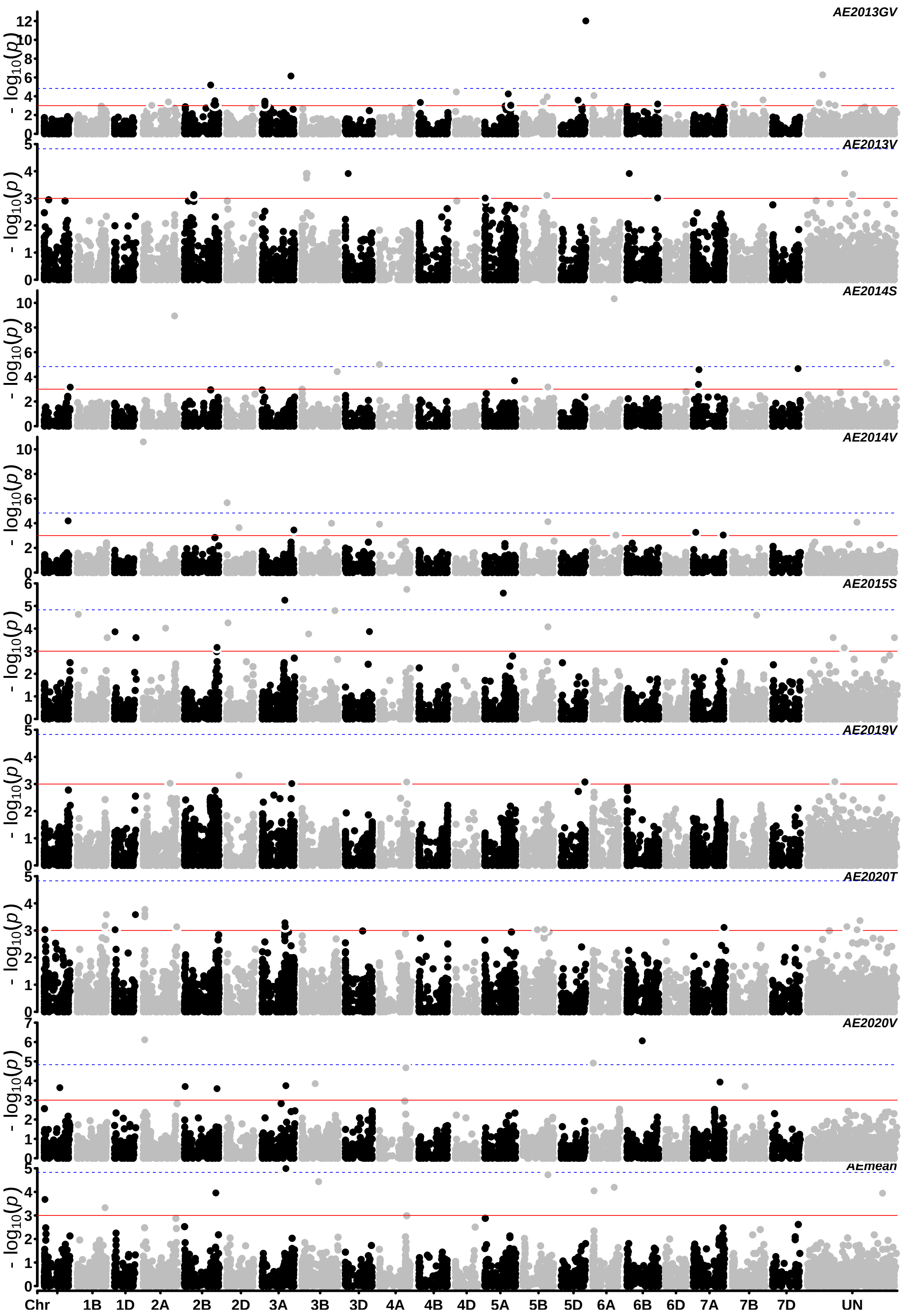
AE2013GV
AE2013V
AE2014S
AE2014V
AE2015S
AE2019V
AE2020T
AE2020V
AEmean
- log10(p)
Chr
1B
1D
2A
2B
2D
3A
3B
3D
4A
4B
4D
5A
5B
5D
6A
6B
6D
7A
7B
7D
UN

(e)

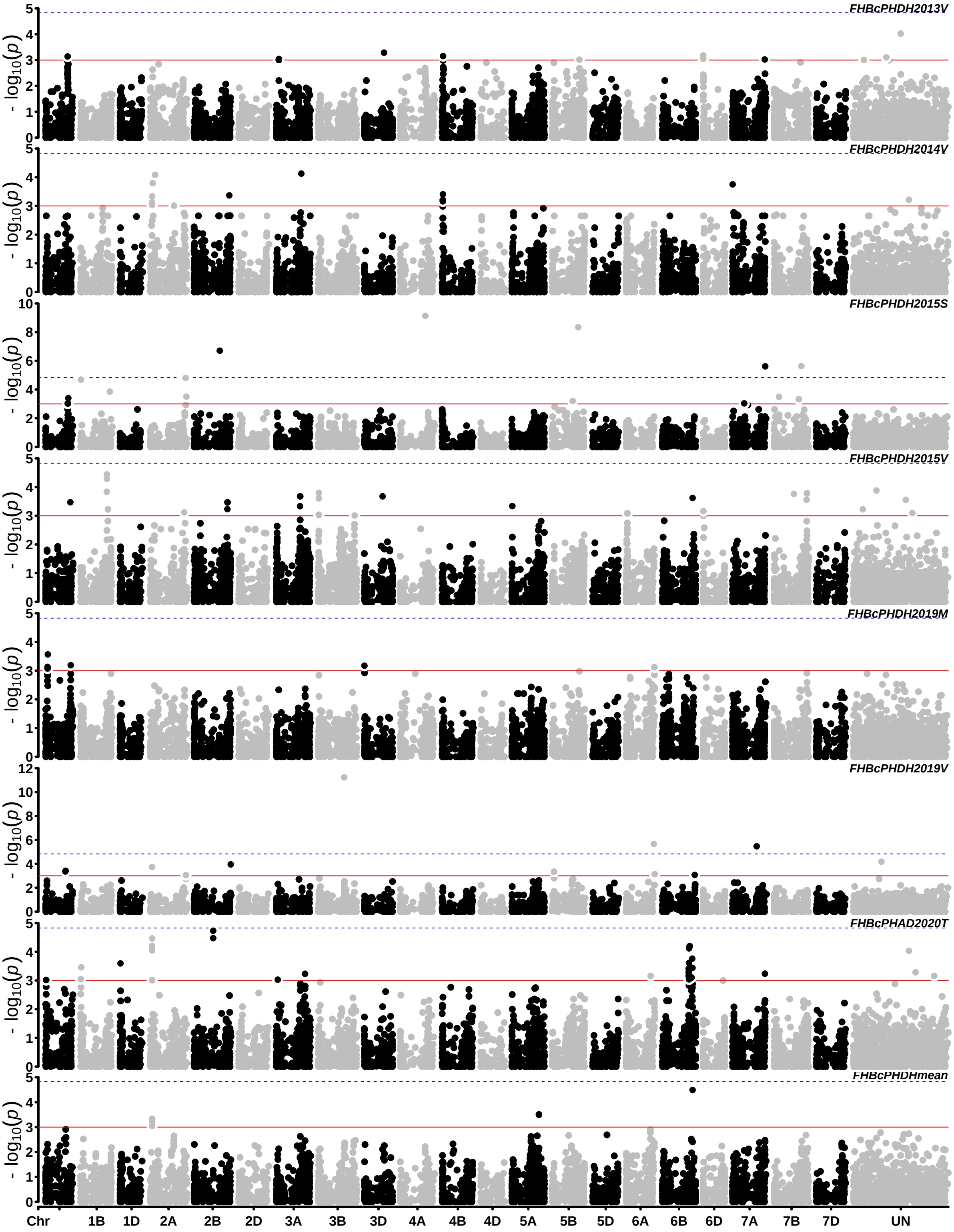

FHBcPHDH2013V
FHBcPHDH2014V
FHBcPHDH2015S
FHBcPHDH2015V
FHBcPHDH2019M
FHBcPHDH2019V
FHBcPHAD2020T
FHBcPHDHmean
- log10(p)
Chr
1B
1D
2A
2B
2D
3A
3B
3D
4A
4B
4D
5A
5B
5D
6A
6B
6D
7A
7B
7D
UN

(f)

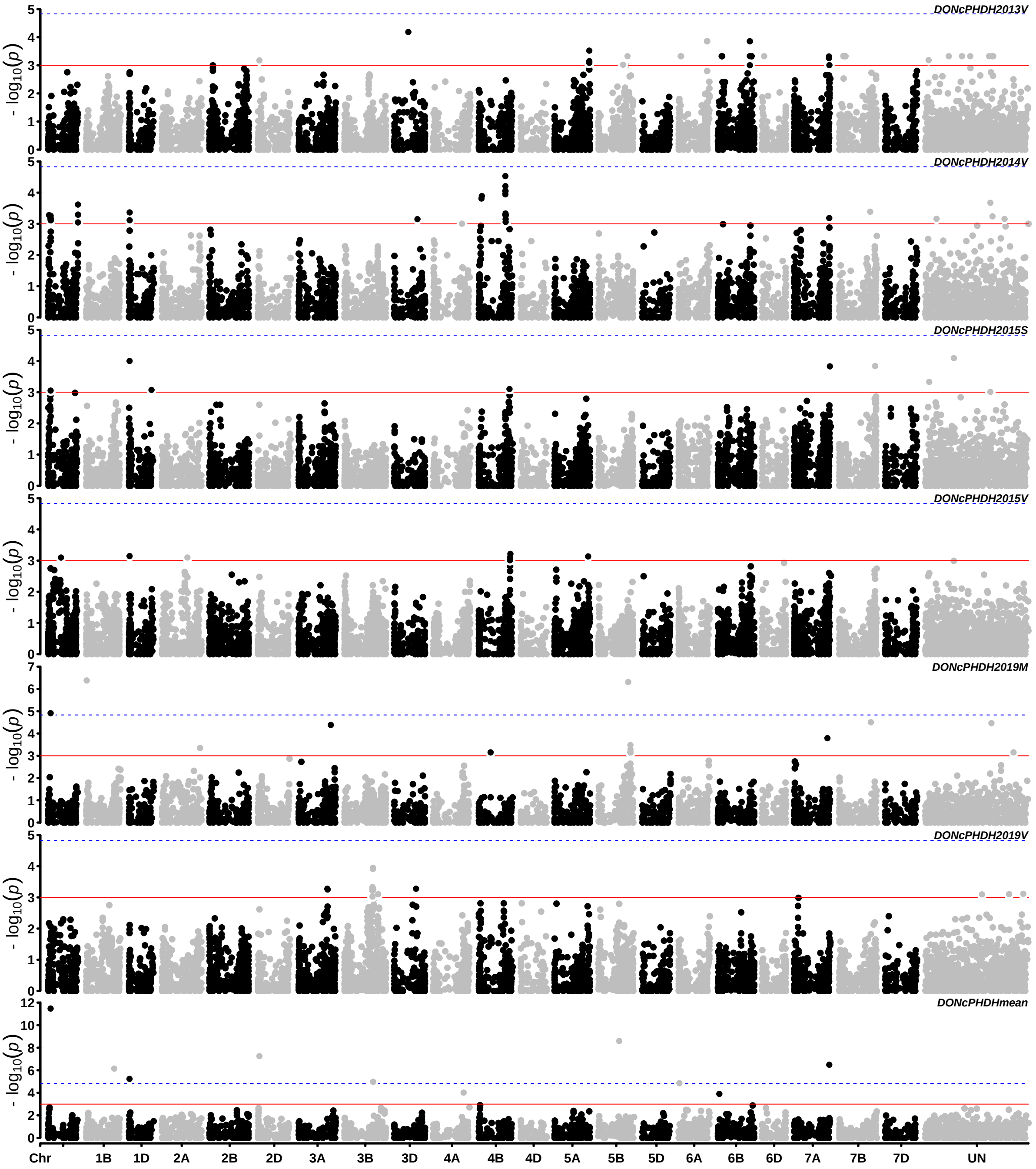

Supplementary figure 5: Manhattan plots of traits AE (a and d), FHBcPHDH (b and e), and DONcPHDH (c and f) in complete panel and European panel respectively for 25K, red line represents significant threshold (-log10p = 3.0) and blue dotted line is FDR @ 0.05% based on across environment means. Suffix to the trait names indicate the names of experimental locations: “GV” for “GreenhouseVollebekk”, “M” for “Morden”, “S” for Staur, “T” for Tulln, and “V” for Vollebekk.

(a)

(b)

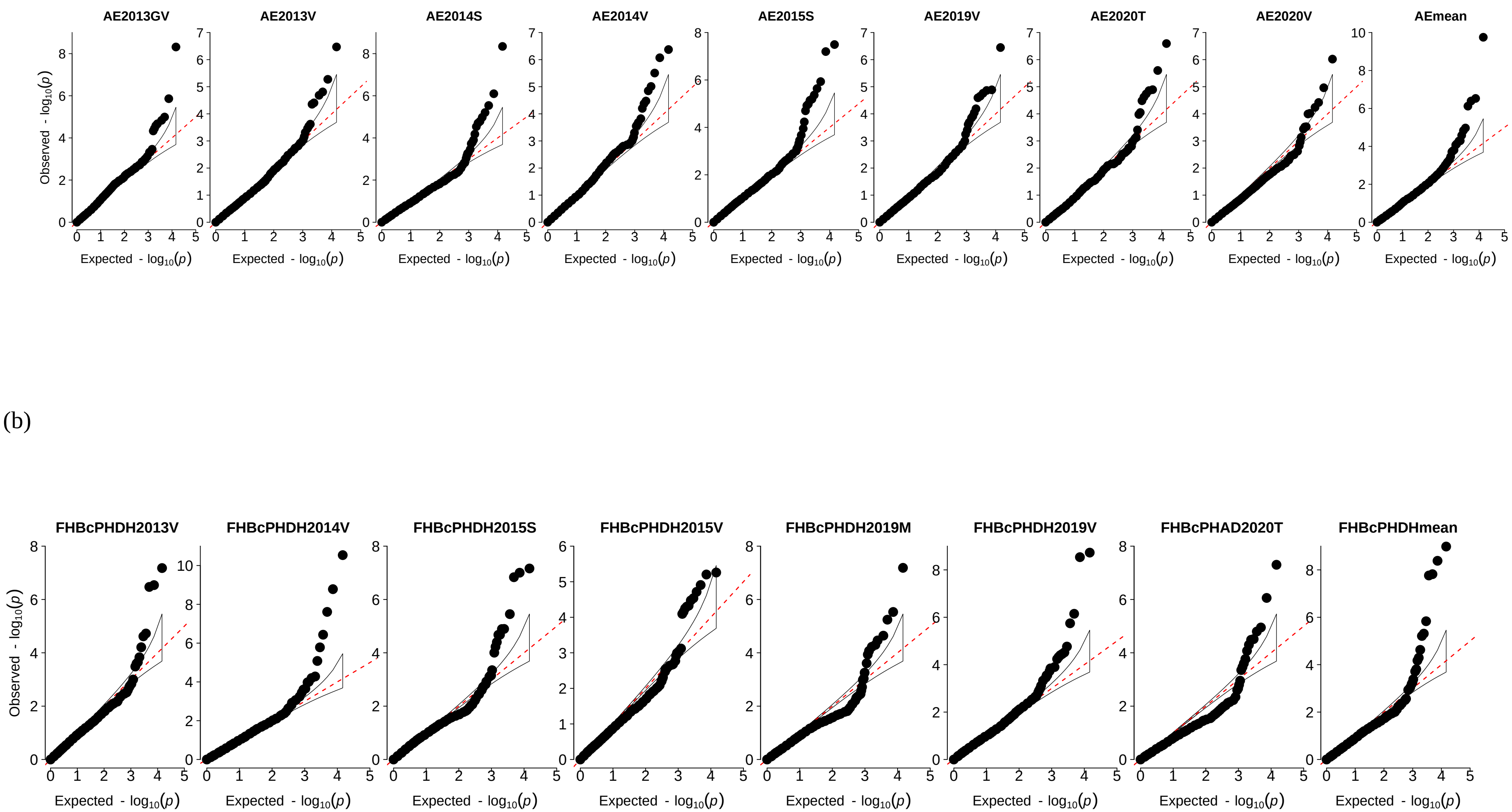
AE2013GV
AE2013V
AE2014S
AE2014V
AE2015S
AE2019V
AE2020T
AE2020V
AEmean
Observed - log10(p)
Expected - log10(p)
FHBcPHDH2013V
FHBcPHDH2014V
FHBcPHDH2015S
FHBcPHDH2015V
FHBcPHDH2019M
FHBcPHDH2019V
FHBcPHAD2020T
FHBcPHDHmean

(c)

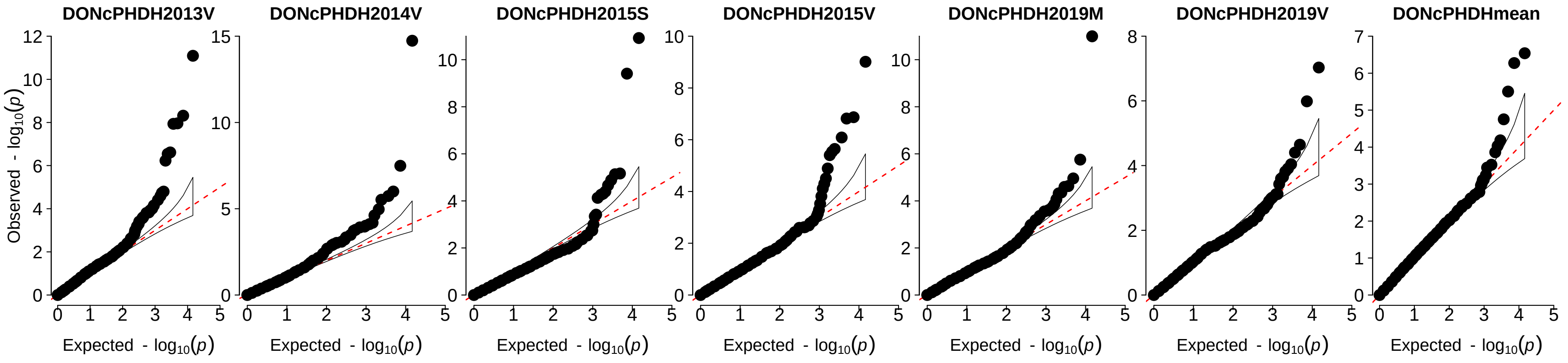
DONcPHDH2013V
DONcPHDH2014V
DONcPHDH2015S
DONcPHDH2015V
DONcPHDH2019M
DONcPHDH2019V
DONcPHDHmean
Observed - $log_{10}(p)$
Expected - $log_{10}(p)$

(d)

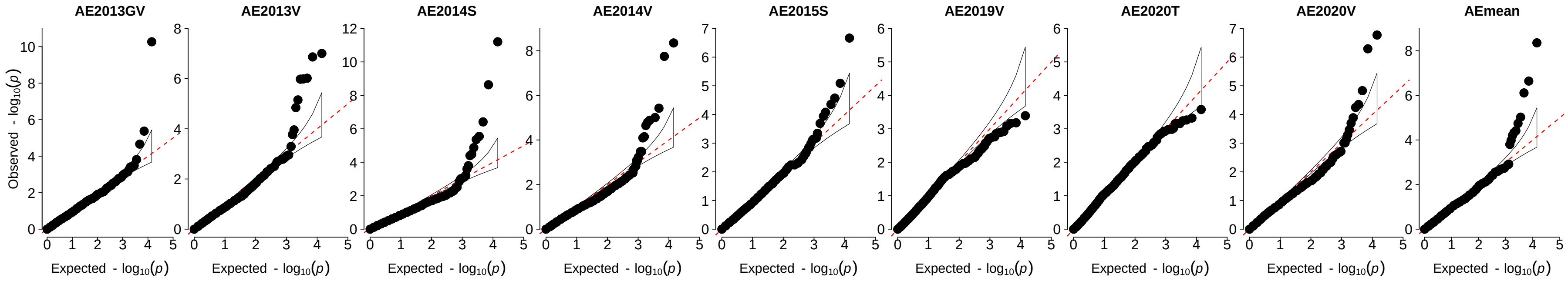
AE2013GV
AE2013V
AE2014S
AE2014V
AE2015S
AE2019V
AE2020T
AE2020V
AEmean
Observed - $log_{10}(p)$
Expected - $log_{10}(p)$

(e)

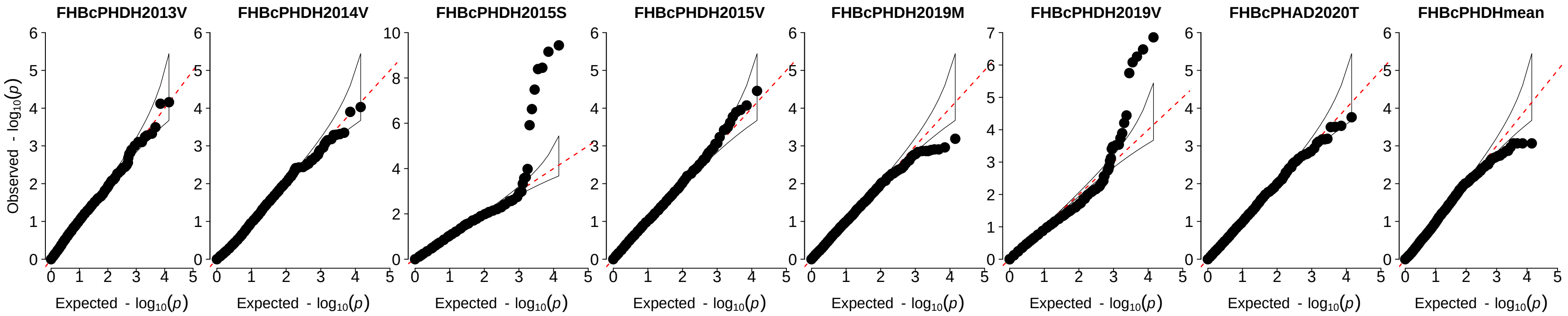

(f)

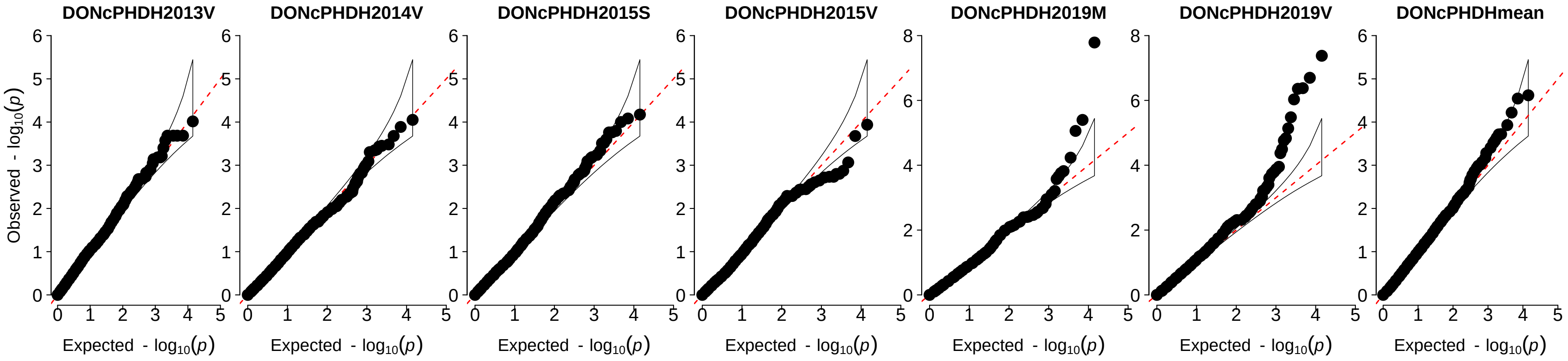

Supplementary figure 6: QQ-plots of traits AE (a and d), FHBcPHDH (b and e), and DONcPHDH (c and f) in complete panel and European panel respectively for 35K, red line represents significant threshold (-log10p = 3.0) and blue dotted line is FDR @ 0.05% based on across environment means. Suffix to the trait names indicate the names of experimental locations: “GV” for “GreenhouseVollebekk”, “M” for “Morden”, “S” for Staur, “T” for Tulln, and “V” for Vollebekk.

(a)

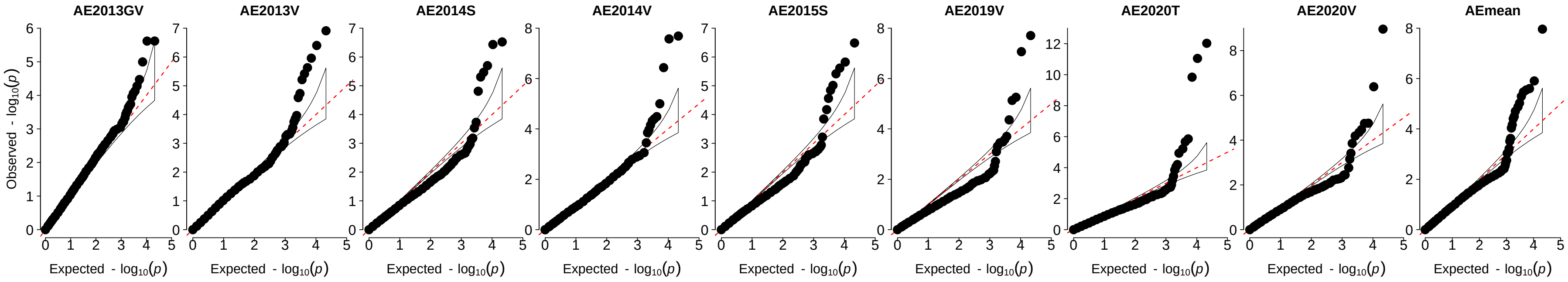
AE2013GV
AE2013V
AE2014S
AE2014V
AE2015S
AE2019V
AE2020T
AE2020V
AEmean
Observed - log10(p)
Expected - log10(p)

(b)

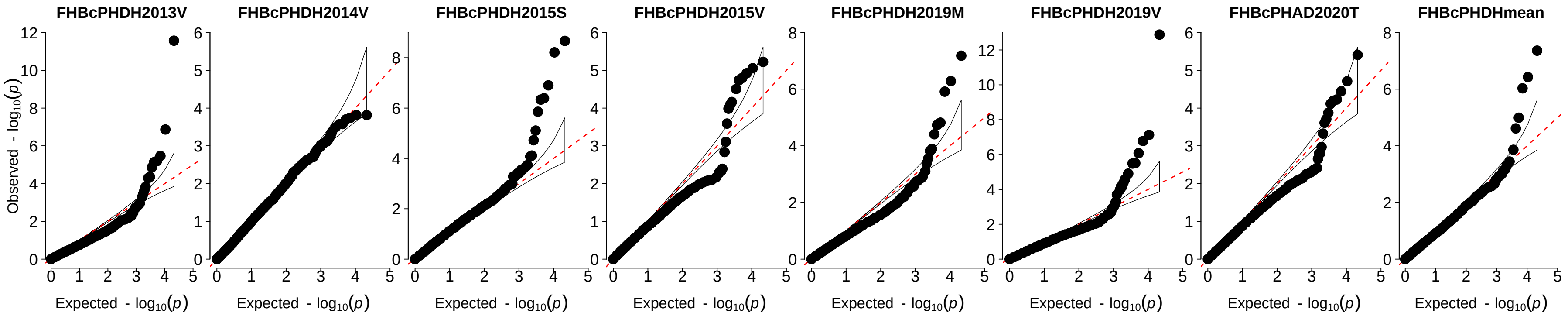
FHBcPHDH2013V
FHBcPHDH2014V
FHBcPHDH2015S
FHBcPHDH2015V
FHBcPHDH2019M
FHBcPHDH2019V
FHBcPHAD2020T
FHBcPHDHmean
Observed - log10(p)
Expected - log10(p)

(c)

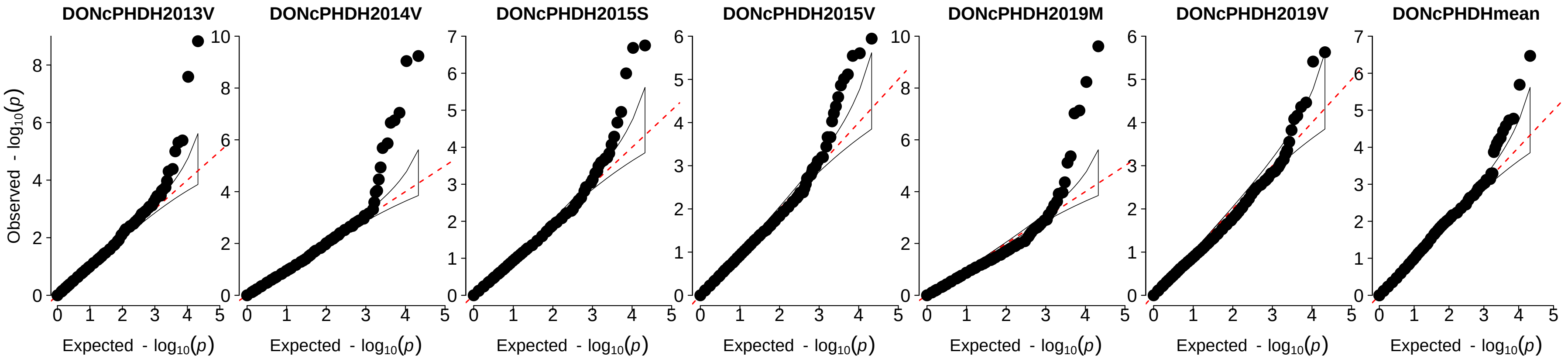

DONcPHDH2013V
DONcPHDH2014V
DONcPHDH2015S
DONcPHDH2015V
DONcPHDH2019M
DONcPHDH2019V
DONcPHDHmean
Observed - log10(p)
Expected - log10(p)

(d)

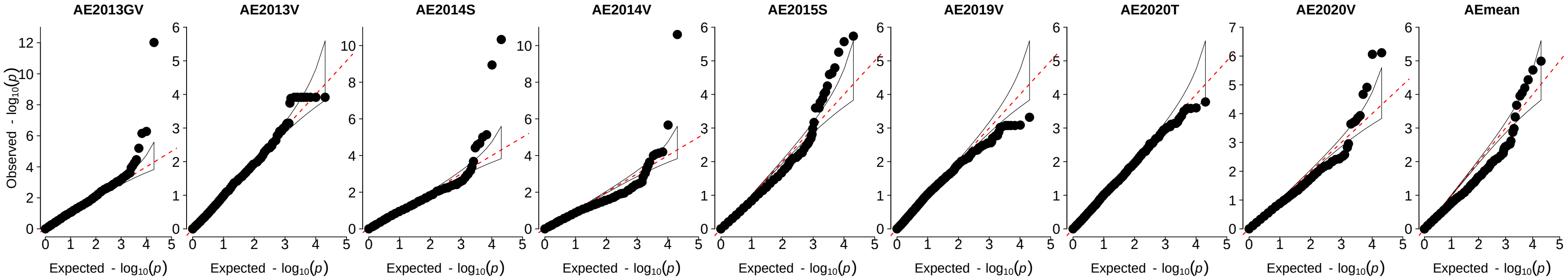

AE2013GV
AE2013V
AE2014S
AE2014V
AE2015S
AE2019V
AE2020T
AE2020V
AEmean
Observed - log10(p)
Expected - log10(p)

(e)

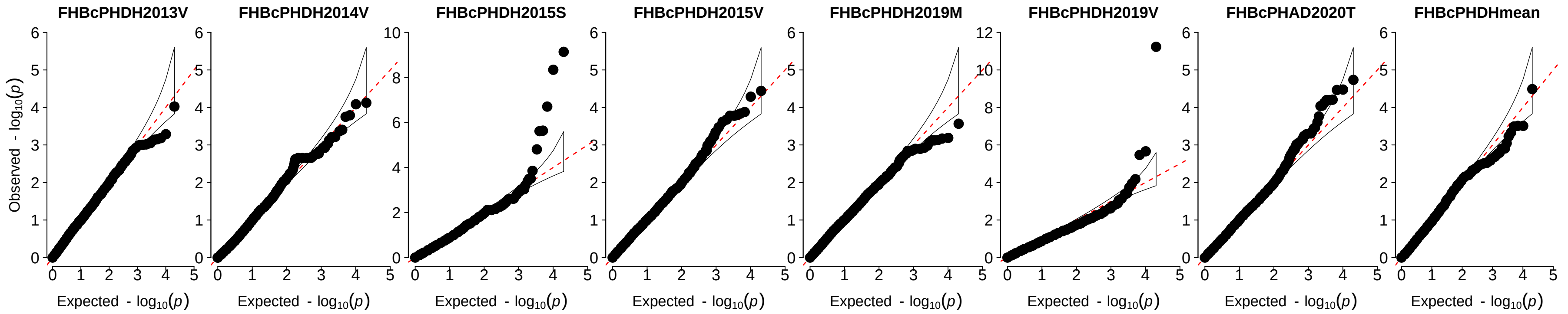

(f)

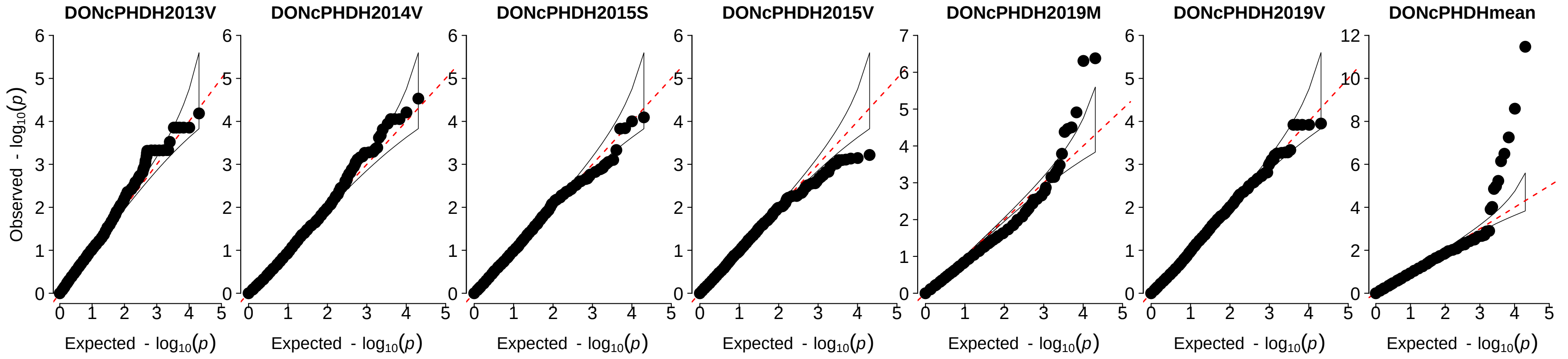

Supplementary figure 7: QQ-plots of traits AE (a and d), FHBcPHDH (b and e), and DONcPHDH (c and f) in complete panel and European panel respectively for 35K, red line represents significant threshold (-log10p = 3.0) and blue dotted line is FDR @ 0.05% based on across environment means. Suffix to the trait names indicate the names of experimental locations: “GV” for “GreenhouseVollebekk”, “M” for “Morden”, “S” for Staur, “T” for Tulln, and “V” for Vollebekk.

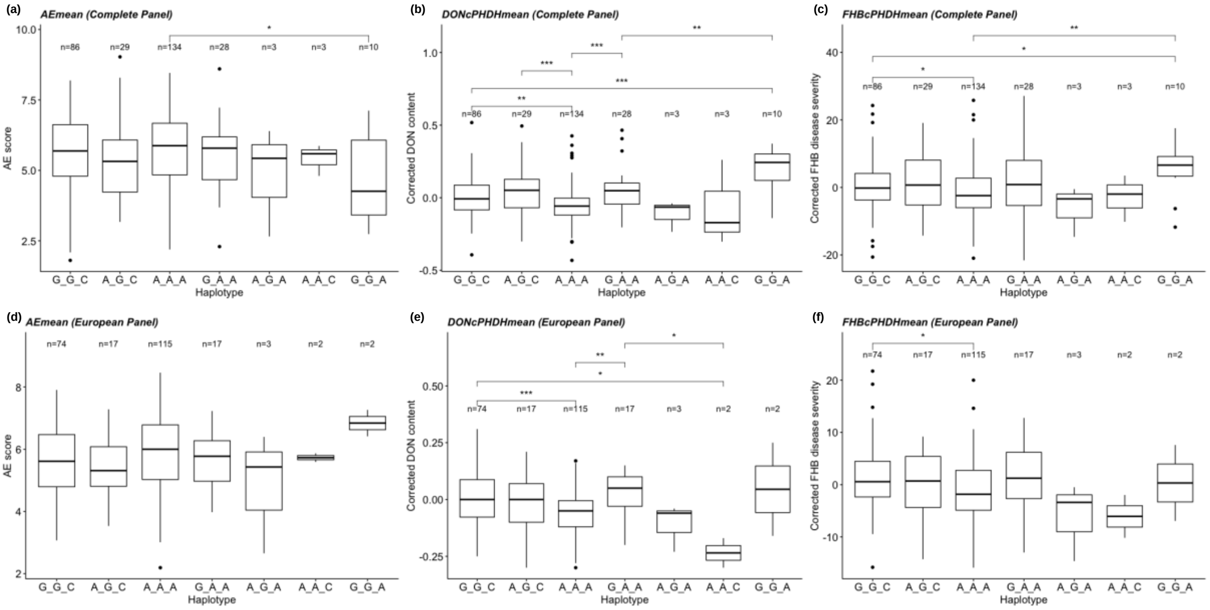

Supplementary figure 8: Boxplot showing the haplotype effect of QTL *Qfhb.nmbu.1A.1* on chromosome 1A based on the a) mean AE, b) mean corrected DON content, c) mean corrected FHB disease severity of complete panel, and d) mean AE, e) mean corrected DON content, f) mean corrected FHB disease severity of European panel. Wilcoxon method is used for pair-wise comparisons (***P < 0.0001, **P < 0.001, *P < 0.05, ns > 0.05). Comparisons with ns are not shown in this figure.

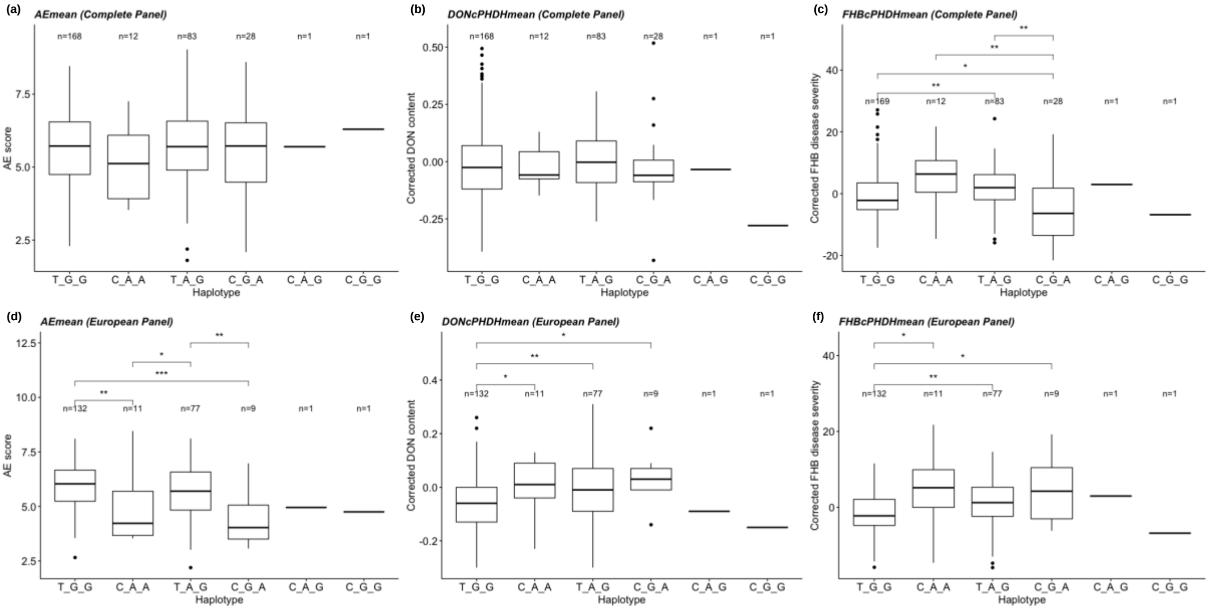

Supplementary figure 9: Boxplot showing the haplotype effect of QTL *Qfhb.nmbu.3A.1* on chromosome 3A based on the a) mean AE, b) mean corrected DON content, c) mean corrected FHB disease severity of complete panel, and d) mean AE, e) mean corrected DON content, f) mean corrected FHB disease severity of European panel. Wilcoxon method is used for pair-wise comparisons (***P < 0.0001, **P < 0.001, *P < 0.05, ns > 0.05). Comparisons with ns are not shown in this figure.

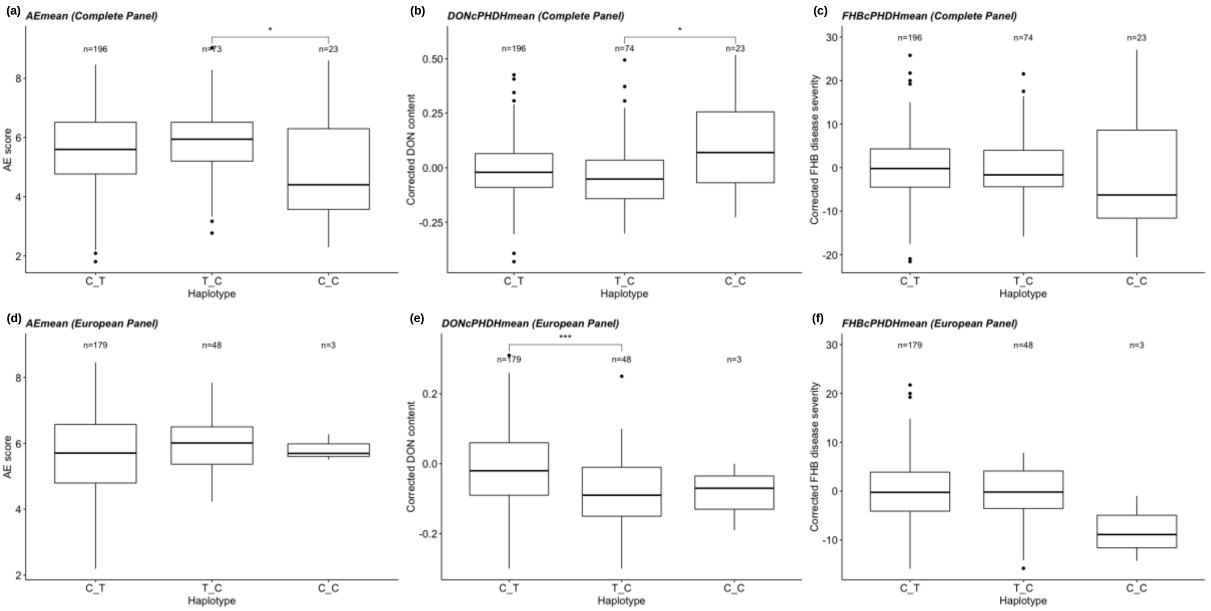

Supplementary figure 10: Boxplot showing the haplotype effect of QTL *Qfhb.nmbu.4B.1* on chromosome 4B based on the a) mean AE, b) mean corrected DON content, c) mean corrected FHB disease severity of complete panel, and d) mean AE, e) mean corrected DON content, f) mean corrected FHB disease severity of European panel. Wilcoxon method is used for pair-wise comparisons (***P < 0.0001, **P < 0.001, *P < 0.05, ns > 0.05). Comparisons with ns are not shown in this figure.

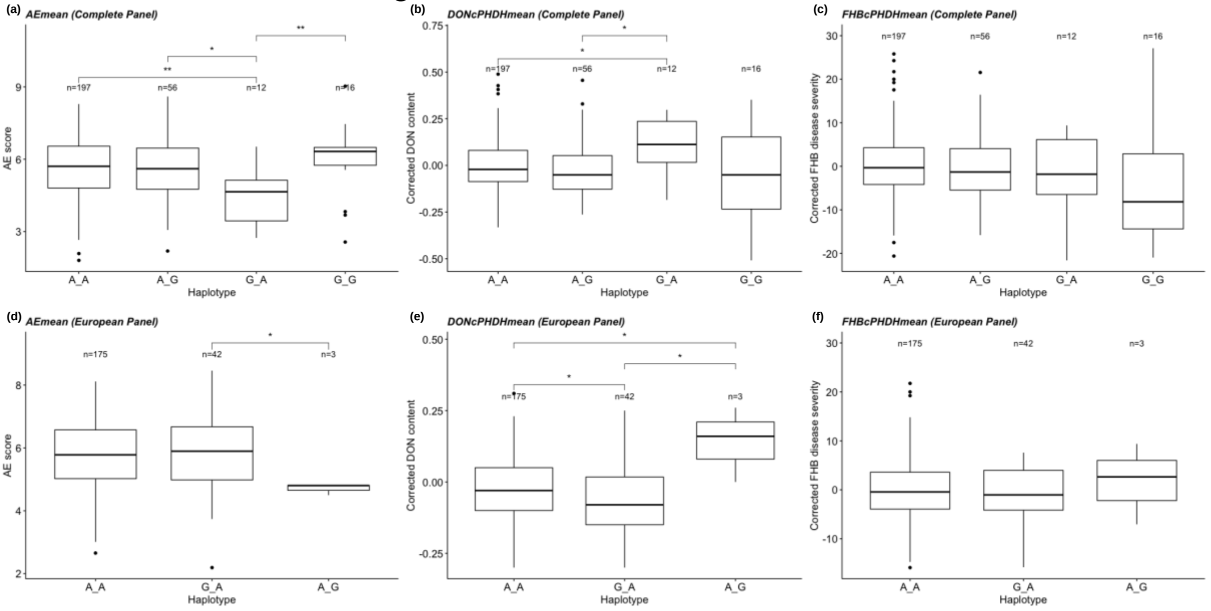

Supplementary figure 11: Boxplot showing the haplotype effect of QTL *Qfhb.nmbu.5A.1* on chromosome 5A based on the a) mean AE, b) mean corrected DON content, c) mean corrected FHB disease severity of complete panel, and d) mean AE, e) mean corrected DON content, f) mean corrected FHB disease severity of European panel. Wilcoxon method is used for pair-wise comparisons (***P < 0.0001, **P < 0.001, *P < 0.05, ns > 0.05). Comparisons with ns are not shown in this figure.

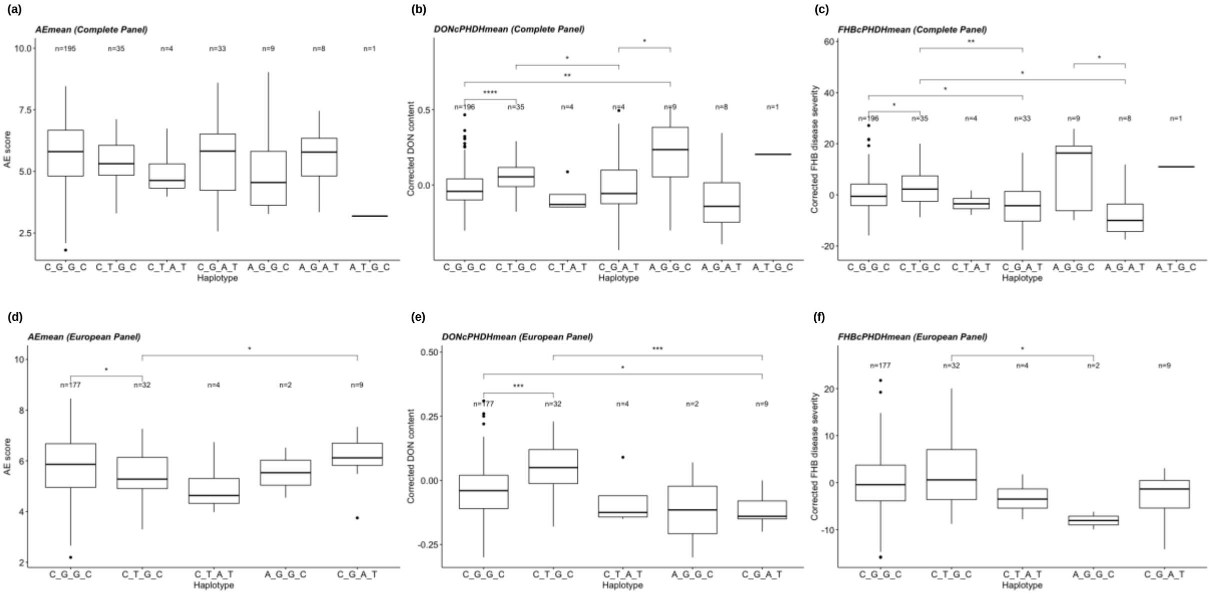

Supplementary figure 12: Boxplot showing the haplotype effect of QTL *Qfhb.nmbu.6A.1* on chromosome 6A based on the a) mean AE, b) mean corrected DON content, c) mean corrected FHB disease severity of complete panel, and d) mean AE, e) mean corrected DON content, f) mean corrected FHB disease severity of European panel. Wilcoxon method is used for pair-wise comparisons (***P < 0.0001, **P < 0.001, *P < 0.05, ns > 0.05). Comparisons with ns are not shown in this figure.

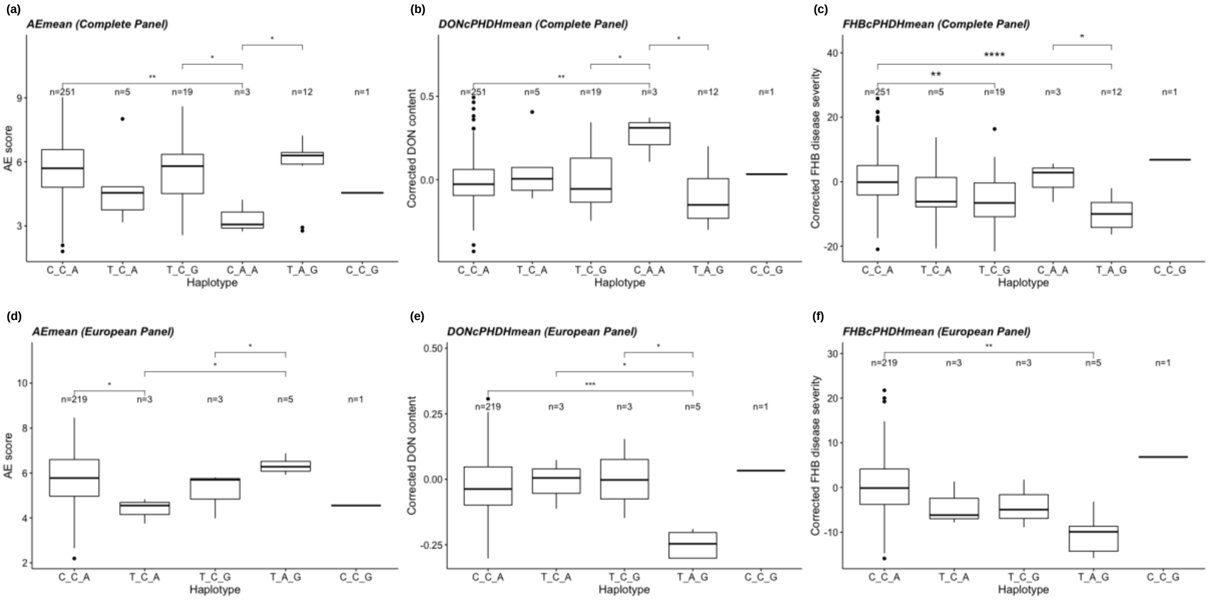

Supplementary figure 13: Boxplot showing the haplotype effect of QTL *Qfhb.nmbu.7A.2* on chromosome 7A with KASP markers based on the a) mean AE, b) mean corrected DON content, c) mean corrected FHB disease severity of complete panel, and d) mean AE, e) mean corrected DON content, f) mean corrected FHB disease severity of European panel. Wilcoxon method is used for pair-wise comparisons (***P < 0.0001, **P < 0.001, *P < 0.05, ns > 0.05). Comparisons with ns are not shown in this figure.

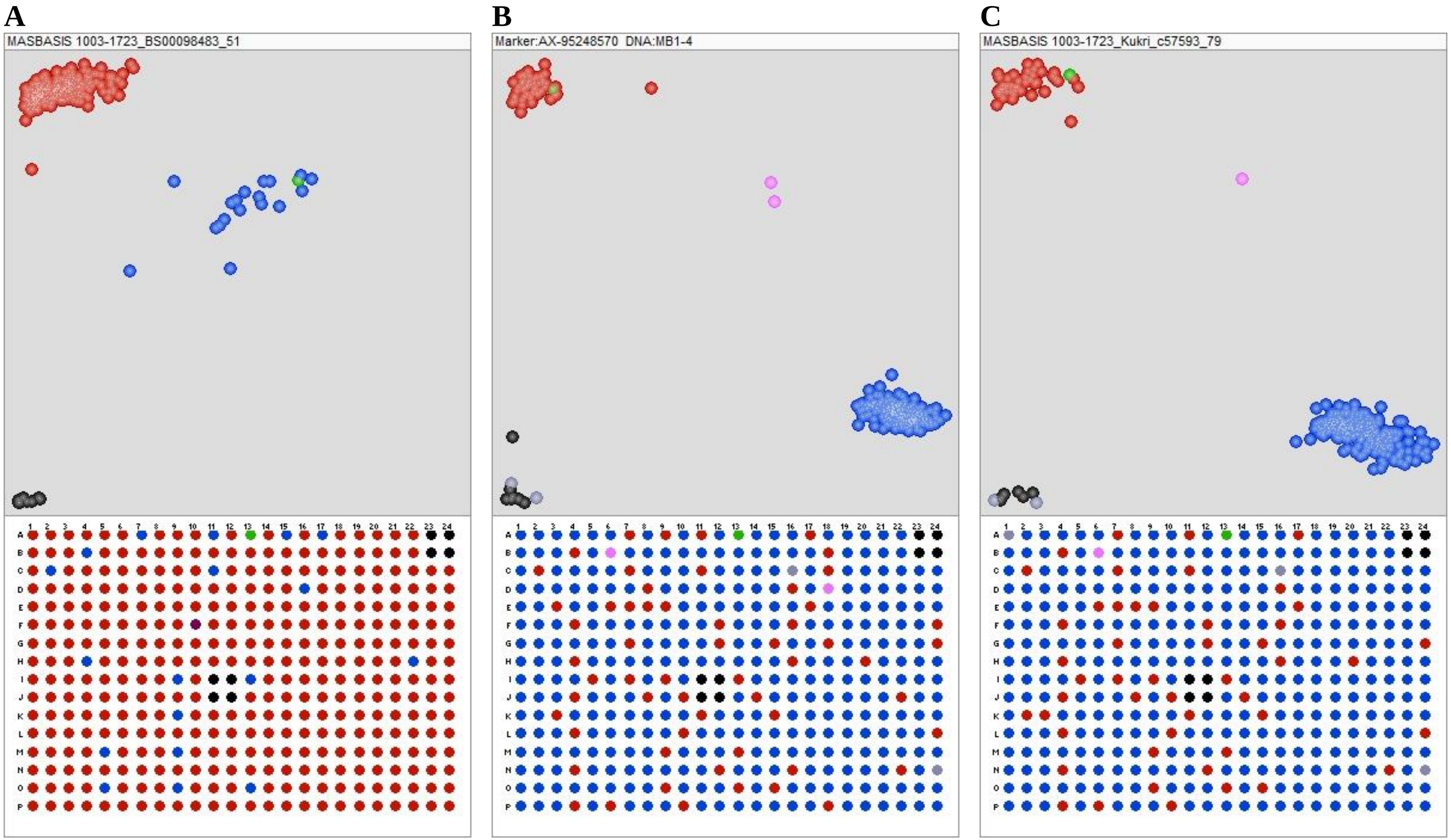

Supplementary figure 14: Cluster plots for the three KASP markers used for genotyping the NMBU spring wheat panel: a) BS00098483_51, b) AX-95248570, and c) Kukri_c57593_79. Genotype calls for allele one (FAM) and two (HEX) are indicated in *blue* and *red*, respectively. Heterozygous scores are shown in *pink* and negative controls in *black*. The source of the resistant allele of *Qfhb.nmbu.7A.2*, Ning8343, for is shown in *green*.
